# Cost-effectiveness of home-based screening of the general population for albuminuria to prevent progression of cardiovascular and kidney disease

**DOI:** 10.1016/j.eclinm.2023.102414

**Published:** 2024-01-17

**Authors:** Xavier G.L.V. Pouwels, Dominique van Mil, Lyanne M. Kieneker, Cornelis Boersma, Ronald W. van Etten, Birgitte Evers-Roeten, Hiddo J.L. Heerspink, Marc H. Hemmelder, Marloes L.P. Langelaan, Marc H.M. Thelen, Ron T. Gansevoort, Hendrik Koffijberg

**Affiliations:** a Health Technology and Services Research Department, Technical Medical Centre, Faculty of Behavioral, Management, and Social Sciences, University of Twente, Enschede, the Netherlands; b Department of Internal Medicine, University Medical Centre Groningen, University of Groningen, Groningen, the Netherlands; c Department of Clinical Pharmacy and Pharmacology, University Medical Centre Groningen, University of Groningen, Groningen, the Netherlands; d Unit of Global Health, Department of Health Science, University Medical Centre Groningen, Groningen, the Netherlands; e Faculty of Management Sciences, Open University, Heerlen, the Netherlands; f Department of Internal Medicine, Amphia Hospital, Breda, the Netherlands; g General Practice Tholos, Zevenbergen, the Netherlands; h The George Institute for Global Health, Sydney, NSW, Australia; i Division of Nephrology, Department of Internal Medicine, Maastricht Universal Medical Center and Cardiovascular Research Institute University Maastricht, Maastricht, the Netherlands; j Result Laboratory for Clinical Chemistry, Amphia Hospital, Breda, the Netherlands; k Radboud University, Nijmegen, the Netherlands

**Keywords:** Chronic kidney disease, Albuminuria, Screening, General population, Cost-effectiveness

## Abstract

**Background:**

Chronic kidney disease (CKD) is often detected late, leading to substantial health loss and high treatment costs. Screening the general population for albuminuria identifies individuals at high risk of kidney events and cardiovascular disease (CVD) who may benefit from early start of preventive interventions. Previous studies on the cost-effectiveness of albuminuria population screening were inconclusive, but were based on survey or cohort data rather than an implementation study, modelled screening as performed by general practitioners rather than home-based screening, and often included only benefits with respect to kidney events. We evaluated the cost-effectiveness of home-based general population screening for increased albuminuria based on real-world data obtained from a prospective implementation study taking into account prevention of CKD as well as CVD events.

**Methods:**

We developed an individual-level simulation model to compare home-based screening using a urine collection device with usual care (no home-based screening) in individuals of the general population aged 45–80, based on the THOMAS study (Towards HOMe-based Albuminuria Screening). Cost-effectiveness was assessed from the Dutch healthcare perspective with a lifetime horizon. The costs of the screening process and benefits of preventing CKD progression (dialysis and kidney transplantation) and CVD events (non-fatal myocardial infarction, non-fatal stroke, fatal CVD event) were reflected. Albuminuria detection led to treatment of identified risk factors. The model subsequently simulated CKD progression, the occurrence of CVD events, and death. The risks of experiencing CVD events were calculated using the SCORE2 CKD risk prediction model and individual-level data from the THOMAS study. Relative treatment effectiveness, quality of life scores, resource use, and cost inputs were obtained from literature. Model outcomes were the number of CKD and CVD-related events, total costs, quality-adjusted life years (QALYs), and the incremental cost-effectiveness ratio (ICER) per QALY gained by screening versus usual care. All results were obtained through probabilistic analysis.

**Findings:**

The absolute difference between screening versus usual care in lifetime probability of dialysis, kidney transplantation, non-fatal myocardial infarction, non-fatal stroke, and fatal CVD events were 0.2%, 0.05%, 0.6%, 0.6%, and 0.2%, respectively. This led to relative decreases compared to usual care in lifetime incidence of these events of 10.7%, 11.1%, 5.1%, 4.1%, and 1.6%, respectively. The incremental costs and QALYs of screening were €1607 and 0.17 QALY, respectively, which led to a corresponding ICER of €9225/QALY. The probability of screening being cost-effective for the Dutch willingness-to-pay threshold for preventive population screening of €20,000/QALY was 95.0%. Implementing the screening in the subgroup of 45–64 years old reduced the ICER (€7946/QALY), whereas implementing screening in the subgroup of 65–80 years old increased the ICER (€10,310/QALY). A scenario analysis assuming treatment optimization in all individuals with newly diagnosed risk factors or known risk factors not within target range reduced the ICER to €7083/QALY, resulting from the incremental costs and QALY gain of €2145 and 0.30, respectively.

**Interpretation:**

Home-based screening for increased albuminuria to prevent CVD and CKD events is likely cost-effective. More health benefits can be obtained by screening younger individuals and better optimization of care in individuals identified with newly diagnosed or known risk factors outside target range.

**Funding:**

10.13039/501100002997Dutch Kidney Foundation, Top Sector Life Sciences & Health of the Dutch Ministry of Economic Affairs.

**Research in context**

**Evidence before this study**

Increased albuminuria defines chronic kidney disease (CKD) independent of kidney function and is a predictor for progressive CKD and cardiovascular disease. Screening the general population for increased albuminuria may provide an opportunity to detect and treat CKD early, thereby preventing CKD and cardiovascular disease progression. We searched PubMed with the terms “chronic kidney disease”, “albuminuria”, “screening”, “cost-effectiveness”, “economic evaluation”, and “general population” for articles published between Jan 1, 2000, and August 1, 2023, in all languages, to identify studies performing a health economic assessment of screening for albuminuria and CKD. These studies suggest that CKD screening is cost-effective when performed in specific high-risk populations, such as patients with type 2 diabetes or hypertension. Evidence for population-wide screening, however, is limited and inconclusive.

**Added value of this study**

Based on the results of our recently published prospective, randomized implementation study (Towards Home-based Albuminuria Screening, THOMAS), we developed a de novo individual-level health-state transition model to assess the cost-effectiveness of home-based albuminuria screening. To our knowledge, this is the first cost-effectiveness analysis for general population CKD screening based on data from a real-world implementation study. Our analysis shows that home-based albuminuria screening among the general population aged 45–80, compared to usual care (no home-based screening), is cost-effective in preventing kidney and cardiovascular events. We found an incremental cost-effectiveness ratio of €9225/QALY, well below commonly applied willingness-to-pay thresholds. Scenario analyses indicated that cost-effectiveness could be further improved by better optimizing the introduction of care in individuals identified as having increased albuminuria.

**Implications of all the available evidence**

There is abundant evidence for the cost-effectiveness of CKD screening in high-risk populations. However, in such a strategy many high-risk subjects are not screened as deemed appropriate, and many subjects at high risk are not known yet as being at high-risk. Our results show the potential of home-based screening of the general population for albuminuria to identify and cost-effectively treat patients with CKD in an early phase of their disease. Future studies should investigate questions relevant to implementing CKD screening, such as the effect of repeat screening, time intervals between repeat screenings, and cost-effectiveness in lower-income countries.

## Introduction

Chronic kidney disease (CKD) is a serious global health problem, that is estimated to affect 700–800 million people around the world.^1^ It is a major risk factor for progression towards kidney failure, with a need for dialysis or a kidney transplant, and for cardiovascular events (CVEs), leading to a high morbidity and mortality.^2^^,^^3^ Consequently, CKD imposes high costs on healthcare systems. In Europe, costs related to CKD are estimated to be around 140 billion euros per year.^4^

Increased albuminuria is a well-established early marker of CKD, as well as a suitable therapeutic target.^3^^,^^5^ A reduction in albuminuria with blood pressure lowering, especially with agents inhibiting the renin-angiotensin system (RAS), is associated with a reduced incidence of kidney events and CVEs in diabetic and hypertensive populations.^6^ RAS inhibition has, therefore, become the cornerstone of CKD treatment, even in normotensive individuals. Recently, clinical trials have shown that adding sodium glucose co-transporter 2 (SGLT2) inhibitors further reduces albuminuria and also improves kidney and CV outcomes. These drugs have, therefore, been added to the standard treatment for CKD.^7^, ^8^, ^9^ Screening for albuminuria may be useful to identify individuals who could benefit from these treatments.

Despite recommendations to perform screening for the detection of CKD in individuals with established risk factors (i.e., hypertension, diabetes, a history of CVE), CKD is vastly underdiagnosed in these subgroups.^4^^,^^10^^,^^11^ Furthermore, many people have hypertension, hypercholesterolemia, or type 2 diabetes, but are not yet diagnosed with these risk factors, and are therefore not screened for CKD.^12^^,^^13^ In addition, CKD can be present in people without these established risk factors. Population-wide screening could be an attractive option to detect these individuals, but evidence on the cost-effectiveness of such a screening is inconclusive, with most, but not all, studies reporting that CKD screening in the general population is not cost-effective.^14^^,^^15^ However, in these cost-effectiveness studies, based on either survey data or cohort studies, screening only aimed to detect severely increased albuminuria (ACR ≥30 mg/mmol) or eGFR <60 ml/min/1.73 m^2^, whereas moderately increased albuminuria (ACR ≥3–30 mg/mmol) is far more common, and also associated with worse outcomes.^16^^,^^17^ Moreover, screening was performed in the general practitioner (GP) setting, which involves high costs. Home-based screening may be more cost-effective. Lastly, often, only treatment benefit with respect to the prevention of kidney failure was taken into account, whereas intervention in individuals with albuminuria will also improve cardiovascular prognosis.^16^, ^17^, ^18^, ^19^

Given these considerations, we aimed to evaluate the cost-effectiveness of a home-based screening of the general population for moderately to severely increased albuminuria to prevent kidney as well as cardiovascular events in the general population from the Dutch healthcare perspective. This evaluation is based on real-world data obtained from a prospective implementation study with benefit modelled for kidney as well as cardiovascular outcomes.

## Methods

A *de novo* individual-level health-state transition model was developed to assess the cost-effectiveness of a home-based albuminuria screening strategy using a urine collection device versus usual care (no home-based screening). The model was informed by the Towards HOMe-based Albuminuria Screening (THOMAS) study (NCT04295889).^20^ This randomized study evaluated the effectiveness of two home-based albuminuria screening strategies in 15,074 Dutch individuals aged 45–80.^21^ Individuals were randomized (1:1) to receive at home a urine collection device to collect some urine, and whereafter this device was sent by regular post to a central laboratory for albumin:creatinine ratio (ACR) measurement or a package containing material and instructions to download a smart-phone app (App) to measure the ACR with a dipstick method at home. Because the App strategy resulted in too many false positive results to be suitable for screening, the present cost-effectiveness study reports only the results obtained using the urine collection device. In case of confirmed increased albuminuria (ACR ≥ 3 mg/mmol in two urine samples collected at home), individuals were invited for an elaborate screening at a central screening facility for CKD and cardiovascular risk factors (blood pressure, cholesterol, glucose, kidney function). In case subjects were diagnosed with or thought that they might have a urinary tract infection, they were asked to postpone the collecting urine. When abnormalities (either newly diagnosed risk factors or known risk factors that were outside target range) were found, individuals were referred to their GP for start or optimization of treatment.

Total life years (LY), quality-adjusted life years (QALYs), costs, and the number of kidney events and CVEs were determined for the home-based screening versus usual care. The incremental cost-effectiveness ratios (ICERs) per QALY gained were calculated by dividing the incremental costs by the incremental QALYs of home-based screening versus usual care. The analysis was performed from the Dutch healthcare perspective, using a lifetime time horizon (60 years) due to the chronic character of CKD and cardiovascular disease. Health outcomes were discounted at 1.5% yearly and costs at 4.0% according to the Dutch guidelines for conducting health economic evaluations.^22^ This analysis is reported in line with the CHEERS 2022 guidelines, and validated using the AdviSHE and TECH-VER tools (Appendix pp 27–32).^23^, ^24^, ^25^

### Ethics statement

Before initiation of the study, the study protocol of THOMAS was approved by the Medical Ethics Committee of the University Medical Center Groningen, Groningen, the Netherlands (2018.687). All participants provided written informed consent prior to participation.

### Population and comparators

The target population consisted of Dutch adults aged 45–80, as included in the THOMAS study, that was recently published in the Lancet.^21^ The health economic (HE) model compared home-based screening as described above with usual care. The following risk factors were considered in this HE model: albuminuria, CKD, hypertension, hyperlipidemia, and heart failure, as measured in the THOMAS study. Albuminuria levels of individuals were determined based on their ACR during the elaborate screening, and CKD stage was determined based on albuminuria level and estimated Glomerular Filtration Rate (eGFR) (Appendix pp 7–8). For albuminuria and CKD, individuals were considered either ‘undiagnosed’, ‘known’, or ‘newly diagnosed’. For hypertension and hyperlipidemia, individuals were categorized as ‘undiagnosed’, ‘known, within target values’, ‘known, outside target values’, or ‘newly diagnosed’. For heart failure, individuals were considered either ‘undiagnosed’ or ‘diagnosed’ before the screening, based on their medical history as reported by the individuals themselves, and were not newly diagnosed with heart failure during the elaborate screening. Only the risk factor profiles of individuals who participated in the elaborate screening were used in the HE model because risk factor levels were only measured during the elaborate screening of the THOMAS study.^21^

### Model overview

**Fig. 1:**
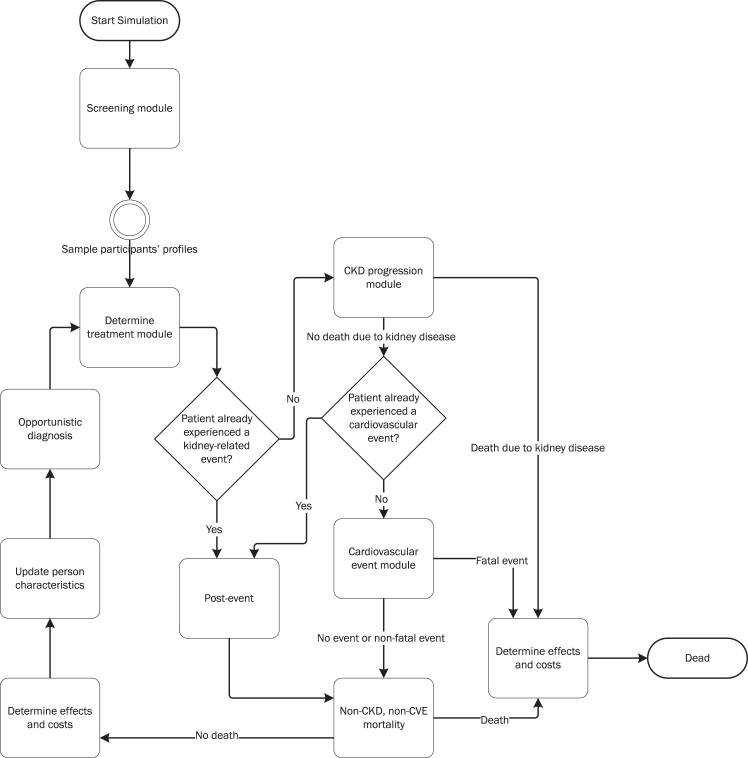
**Simulation model process**. Abbreviations: CKD, chronic kidney disease; CVE, cardiovascular event.

The model structure was informed by previous HE analyses for CKD treatment and screening and developed in collaboration with a clinical expert (RTG) (Fig. 1, Appendix pp 5–12).^26^, ^27^, ^28^, ^29^ The model first simulated the screening process to determine which individuals participated in the elaborate screening and entered the simulation, and which individuals did not due to no participation or negative test results. Thus, response and non-response probabilities are considered in the model. After simulating the screening process, the model used time cycles (steps) of one year. During each time cycle, individuals could transition from one health state to another. In the model, after treatment initiation (1st cycle) or discontinuation, opportunistic diagnosis of undiagnosed risk factors, CKD development and progression (until dialysis and kidney transplantation), the occurrence of CVEs, and death were simulated. The included CVEs were non-fatal myocardial infarction (MI), non-fatal stroke, and fatal CVEs as defined in the SCORE2 prediction model.^30^ Individuals who experienced a non-fatal MI or stroke could still experience a kidney-related event. Individuals who experienced a kidney-related event could not experience a cardiovascular event anymore. This choice was made because of the complex structure of the HE model and to avoid the need for even more additional assumptions. In all cycles, individuals could die from non-CKD and non-CVE related causes according to sex- and age-specific Dutch general population mortality rates (Appendix p 22).

### Model assumptions

We made the following assumptions to populate the HE model for the base-case analyses:

1. In the home-based screening strategy, the treatment of individuals visiting their GP after referral was optimized. This meant that in case of a newly diagnosed risk factor, treatment was started and that in case of a known risk factor that was not within target range for treatment, such treatment was optimized.
2. In the home-based screening strategy, individuals with known risk factors, outside target values for treatment, who did not visit their GP experienced less treatment effectiveness. This was arbitrarily set at half of the treatment effectiveness (50% reduction from the literature estimates for treatment effectiveness).
3. In the usual care strategy, the treatment of individuals with known risk factors, outside target values was not optimized. The reduction in treatment effectiveness was arbitrarily set at half of the treatment effectiveness.
4. Individuals with undiagnosed risk factors were diagnosed opportunistically and initiated treatment. This was arbitrarily set at ten years after the screening.
5. Individuals ≥85 years old could not receive dialysis, individuals ≥75 years old could not undergo kidney transplantation, and 50% of the individuals <75 years old directly underwent kidney transplantation; the remaining individuals underwent dialysis for three years before receiving a kidney transplantation.^31^
6. Treatments allocated to individuals were determined by their diagnosed risk factors. Individuals diagnosed with severely or moderately increased albuminuria with hypertension received an angiotensin-converting enzyme (ACE) inhibitor and an SGLT-2 inhibitor. Individuals diagnosed with hypertension only received an ACE inhibitor. Individuals diagnosed with hyperlipidemia received a statin. Individuals with heart failure received an ACE inhibitor, an SGLT-2 inhibitor, a diuretic, and a beta-blocker. Treatments either reduced CKD progression, the probability of experiencing a CVE, or both.
7. Yearly treatment discontinuation of 3%, based on expert opinion (RG). Individuals discontinuing treatment did not experience any benefits from treatment anymore.

### Model inputs

A synthetic (hypothetical) cohort of 100,000 individuals based on the individual-level data from the 124 participants in the elaborate screening of the THOMAS study was generated, by synthesizing variables one-by-one using sequential regression modelling, to populate the HE model.^21^ This means that each variable (a characteristic of the participant) of the synthetic cohort was estimated based on the previously synthesized variable. This method ensures overlap in the data structure between the original and synthetic cohorts.^32^ We simulated 3.5 million invited individuals per iteration to obtain stable HE results.

Probabilities of participating in each step of the screening program (home-based screening, elaborate screening, and visiting the GP after referral) and of positive results were based on the THOMAS study (Appendix p 13).^21^ The probabilities of albuminuria progression were based on a previous Dutch HE model, using data from the observational PREVEND cohort (Appendix pp 14–16).^29^ SCORE2, adjusted for the CKD risk factor add-on, applying the European low risk region, was used to determine the probability of experiencing CVEs (Appendix pp 17–19).^30^ Treatment effectiveness estimates were based on a meta-analysis and a recent randomized controlled trial (Appendix p 20).^8^^,^^33^ Utility values, which are estimates of the health-related quality of life, were obtained from international literature and were capped to the Dutch general population value (Appendix p 23).

The costs of treatments were obtained from the National Health Care Institute (Appendix pp 24–25).^34^ Screening costs per individual contained the costs of screening tests, elaborate screening, and GP visits. Unit prices for the screening device, sending the device, and measurement of ACR were obtained from the THOMAS study and clinical chemistry laboratory involved. Costs associated with kidney and cardiovascular health states and events were obtained from studies performed in the Dutch context.^35^, ^36^, ^37^ All costs were expressed in euros 2020 and adjusted using the Consumer Price Index if necessary.^38^

### Statistical analysis

All results were obtained from probabilistic analyses of 2000 iterations, using Monte Carlo simulation in R (version 4.0.3).

Probabilities and utility values were assigned beta distributions, costs gamma distributions, and hazard ratios and relative risks lognormal distributions. Daily costs of treatments and costs of (sending and analyzing) the screening device were fixed. Probabilistic estimates of the CVE risk distributions were obtained through bootstrapping the synthetic cohort.

The probabilistic results were plotted in an incremental cost-effectiveness plane. Cost-effectiveness acceptability curves were plotted to display the probability that each strategy was cost-effective at different willingness-to-pay thresholds. The willingness-to-pay thresholds represent the societal value, in euros, of a QALY. Home-based screening was considered cost-effective when its ICER versus usual care would be lower than a willingness-to-pay threshold of €20,000 per QALY, as recommended by the Dutch guidelines for preventive population screening.^39^

We performed five scenario analyses to investigate the impact of several alternative inputs and assumptions on the cost-effectiveness results:

1. Increasing the yearly discontinuation rate of treatment from 3% (base case) to 5%;
2. Increasing the yearly discontinuation rate of treatment from 3% (base case) to 10%;
3. Individuals with known risk factors, outside target values, experienced full treatment effectiveness;
4. Time to opportunistic diagnosis of undiagnosed risk factors was prolonged from ten to 15 years;
5. Time to opportunistic diagnosis of undiagnosed risk factors was shortened from ten to five years;
6. Decreasing the treatment optimization rate in individuals visiting their GP after referral from 100% to 66%;
7. All individuals with known risk factors, outside target values, and newly diagnosed risk factors went to the GP after referral and experienced full treatment effectiveness.

We performed subgroup analyses by stratifying for age. Cost-effectiveness of the home-based screening strategy was compared with usual care in 40–64 and 65–80 years old subgroups separately. We performed one-way sensitivity analyses using ranges of several input variables based on expert opinion to identify the main drivers of the model outcomes. These input variables were: participation probability in home-based screening (set at 50–70%); participation probability in elaborate screening (set at 75–90%); rate of visiting the GP after referral (set at 45–70%); treatment efficacy ( ± 5%); costs of home-based screening ( ± 25%); and costs of SGLT-2 inhibitor treatment ( ± 25%).

A one-year budget impact analysis was performed to estimate the additional costs of implementing the one-time home-based screening strategy and initiating/optimization of treatment in newly diagnosed individuals who visited the GP versus usual care in the 45–80 year old Dutch population in 2021.^40^ The analysis included both screening and treatment costs. The costs of each step of the screening process were obtained by multiplying the price of each step by the number of individuals participating in the specific screening step. Individuals with known risk factors during the elaborate screening were assumed to continue treatment.

### Role of the funding source

The funders of the study had no role in study design, data collection, data analysis, data interpretation, writing of the report, or the decision to submit. All authors had full access to the data. XGLVP, DvM, LMK, RTG, and HK directly accessed and verified the underlying data. All authors took the responsibility for the decision to submit for publication. The final decision for submitting the manuscript was made by XGLVP, DvM, LMK, RTG, and HK.

## Results

### Cohort characteristics

In the THOMAS study, 7522 individuals were invited for home-based albuminuria screening with the urine collection device.^21^ 4484 (59.4%) participated in the home-based screening, of which 150 (3.3%) had confirmed increased albuminuria. 124 of the 150 attended the elaborate screening (82.7%), of whom the individual level-data were the basis for the synthetic cohort. In those individuals, albuminuria, hypertension, hypercholesterolemia, and decreased kidney function (eGFR <60 ml/min/1.73 m^2^) were newly diagnosed in 77 (62.1%), 44 (35.5%), 30 (24.2%), and 27 (21.7%) respectively (Table 1). In total, 89.5% of these individuals (111/124) were referred to their GP because of newly diagnosed risk factors or risk factors outside target range. The generated synthetic cohort of 100,000 individuals resembles the 124 participants of the elaborate screening (Table 1).

**Table 1:** Characteristics of the original cohort (participants of the elaborate screening in the THOMAS study) and matching characteristics of the generated synthetic cohort.

	Original cohort (n = 124)	Synthetic data (n = 100.000)
**Demographics**		
Men	56.5%	56.6%
Age	68.5 (8.9)	68.5 (9.9)
Current smokers	19.4%	20.1%
**Risk factors for progression of CKD and CVD detected during elaborate screening**		
Albuminuria	91.1%	93.8%
Newly diagnosed	54.0%	59.0%
Known	37.1%	34.8%
Decreased eGFR	29.9%	28.8%
Newly diagnosed	21.7%	16.6%
Known	7.3%	11.7%
Type 2 diabetes	31.5%	35.4%
Newly diagnosed	2.4%	3.3%
Known, outside target range	20.2%	21.4%
Known, within target range	8.9%	10.6%
Hypertension	89.5%	90.0%
Newly diagnosed	35.5%	31.1%
Known, outside target range	40.3%	37.6%
Known, within target range	13.7%	21.3%
Hypercholesterolemia	76.6%	73.3%
Newly diagnosed	24.2%	16.8%
Known, outside target range	9.7%	13.0%
Known, within target range	38.7%	43.5%
Heart failure, known	13.7%	14.4%

Data are % or mean (SD). CKD = chronic kidney disease. CVD = cardiovascular disease.

### Cost-effectiveness of home-based albuminuria screening

In the base-case analysis, simulated individuals of home-based albuminuria screening experienced lower lifetime rates of dialysis and kidney transplantation. Screening for home-based albuminuria reduced the lifetime probability of dialysis and kidney transplantation by 0.2% and 0.05%, respectively (Table 2). This led to relative decreases of 10.7% and 11.1% for dialysis and kidney transplantation, respectively, of home-based screening versus usual care. Additionally, over the lifetime horizon, the probability of non-fatal MIs, non-fatal strokes, and fatal CVE decreased by 0.6%, 0.6%, and 0.2%, respectively, for home-based screening compared to usual care. The relative reduction in lifetime non-fatal MIs, non-fatal strokes, and fatal CVE was respectively 5.1%, 4.1%, and 1.6%.

**Table 2:** Number and probability of kidney and cardiovascular events per strategy, expressed per 100.000 individuals participating in the elaborate screening after identification of albuminuria by home-based screening.

	Usual Care	Screening	Absolute difference	Relative difference
**Non-fatal myocardial infarction**				
N	12,080	11,464	−616	−5.1%
Probability	12.1%	11.5%	−0.6%	
**Non-fatal stroke**				
N	14,035	13,463	−572	−4.1%
Probability	14.0%	13.5%	−0.6%	
**Fatal cardiovascular event**				
N	10,541	10,378	−163	−1.6%
Probability	10.5%	10.4%	−0.2%	
**Dialysis**				
N	1799	1606	−193	−10.7%
Probability	1.8%	1.6%	−0.2%	
**Kidney transplantation**				
N	485	431	−54	−11.1%
Probability	0.5%	0.4%	−0.05%	

N = number.

**Fig. 2:**
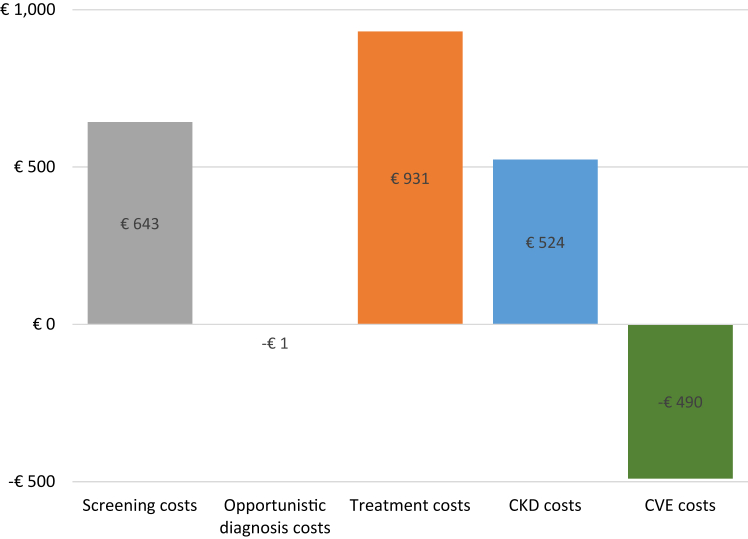
**Incremental costs of home-based albuminuria screening versus usual care per cost category**. Positive costs are in favor of usual care; negative costs are in favor of home-based albuminuria screening. Abbreviations: CKD, chronic kidney disease; CVE, cardiovascular event.

For a one-time home-based screening for albuminuria, the total average survival was estimated at 9.14 LY and for usual care at 8.95 LY. The total QALY gain was estimated at 7.51 QALYs and for usual care at 7.34 QALYs, resulting in an absolute difference of screening versus no screening of 0.17 (Table 3). The incremental mean costs of home-based albuminuria screening versus usual care were estimated at €1607 per invitee over a lifetime. The incremental mean costs of screening were mainly driven by treatment costs (€931) and screening costs (€643), followed by costs for CKD healthcare (€524) (Fig. 2).

**Table 3:** Cost-effectiveness of home-based screening versus usual care for the base-case, scenario, and subgroup analyses.

	Life years	QALY	Total costs	Incremental QALY	Incremental costs	ICER/QALY
**Base-case analysis**						
Usual care	8.95	7.34	€15,236			
Home-based albuminuria screening	9.14	7.51	€16,843	0.17	€1607	€9225
**Scenario analyses**						
Treatment adherence decreases by 5% yearly						
Usual care	8.93	7.32	€15,248			
Home-based albuminuria screening	9.10	7.48	€16,830	0.16	€1582	€10,025
Treatment adherence decreases by 10% yearly						
Usual care	8.89	7.29	€15,268			
Home-based albuminuria screening	9.04	7.42	€16,803	0.13	€1534	€12,005
Individuals with known risk factors, outside target range, experience full treatment effectiveness in all strategies						
Usual care	9.047	7.44	€14,710			
Home-based albuminuria screening	9.25	7.62	€16,381	0.18	€1671	€9307
Opportunistic diagnosis takes place after 15 years in all strategies						
Usual care	8.92	7.31	€15,072			
Home-based albuminuria screening	9.12	7.50	€16,744	0.19	€1672	€8967
Opportunistic diagnosis takes place after 5 years in all strategies						
Usual care	9.01	7.39	€15,636			
Home-based albuminuria screening	9.17	7.54	€17,021	0.14	€1385	€9629
Treatment is optimized in 66% of the individuals visiting the GP upon referral						
Usual care	8.95	7.34	€15,236			
Home-based albuminuria screening	9.04	7.43	€17,027	0.09	€1791	€20,623
All individuals with newly diagnosed risk factors or known but outside target range go to the GP upon referral in the UCD strategy						
Usual care	8.95	7.34	€15,236			
Home-based albuminuria screening	9.04	7.64	€17,381	0.30	€2145	€7083
**Subgroup analyses**						
Subgroup 45–64 years						
Usual care	14.46	11.83	€18,693			
Home-based albuminuria screening	14.77	12.10	€20,847	0.27	€2154	€7946
Subgroup 65–80 years						
Usual care	6.78	5.59	€13,876			
Home-based albuminuria screening	6.93	5.72	€15,277	0.15	€1401	€10,310

QALY = quality-adjusted life years. ICER = incremental cost-effectiveness ratio. GP = general practitioner.

**Fig. 3:**
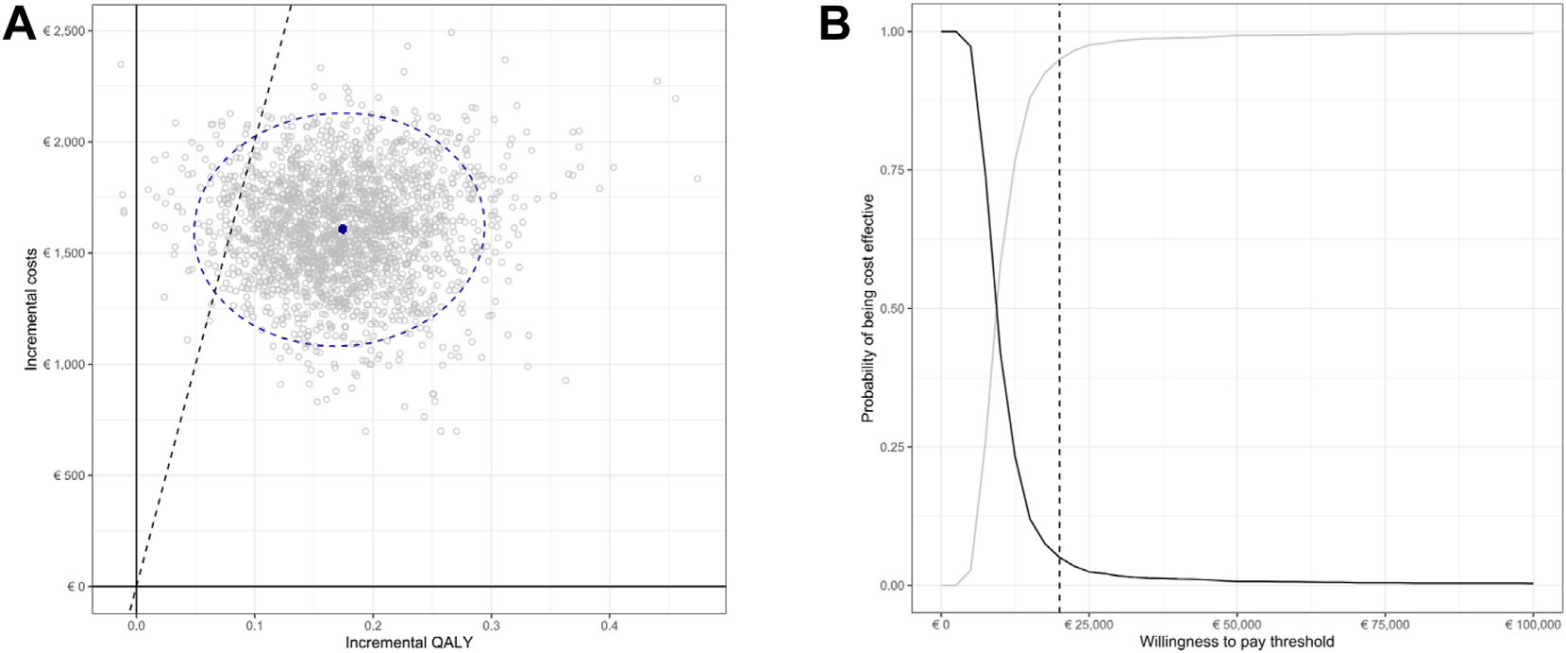
**A. Incremental cost-effectiveness (CE) plane with a willingness-to-pay threshold (dashed line) of €20,000/QALY gained**. This figure indicates that home-based screening, compared to usual care, is likely to be cost-effective at the threshold of €20,000/QALY for most combinations of input values (blue ellipse = 95% confidence ellipse with mean). Abbreviations: QALY = quality-adjusted life years. **B. Cost-effectiveness acceptability curve showing the probability of home-based albuminuria screening being cost-effective at a given willingness-to-pay threshold versus usual care.** The vertical dotted line represents the €20,000/QALY willingness-to-pay threshold. The black line depicts the usual care strategy, the grey line depicts the home-based albuminuria screening strategy.

The incremental gains in QALY and the incremental costs resulted in an ICER of €9225/QALY (Table 3). As depicted in Fig. 3A, home-based albuminuria screening is likely to be cost-effective at a willingness-to-pay threshold of €20,000/QALY for the majority of the combinations of input values. The screening would be cost-effective at a probability of 95.0% for this willingness-to-pay threshold (Fig. 3B).

### Sensitivity analyses

Overall, all performed scenario analyses resulted in home-based albuminuria screening being cost-effective at a willingness-to-pay threshold of €20,000 (Table 3). Assuming a higher decrease in treatment adherence of 10% yearly resulted in the least optimistic results. This scenario decreased the incremental QALYs to 0.13, but also decreased the incremental costs to €1534, resulting in an ICER of €12,005/QALY of home-based albuminuria screening. Assuming individuals with known risk factors, but outside target range, experience full treatment effectiveness, had minimal effect on the ICER. Changing the time to opportunistic diagnosis of risk factors to 15 or five years, respectively, instead of ten years, did not markedly influence the health economic outcomes compared to the base-case analysis (opportunistic diagnosis after ten years) (Table 3).

On the other hand, assuming as an alternative scenario that all individuals would visit their GP and undergo treatment optimization if applicable, did increase the incremental QALYs to 0.30 and costs obtained by screening to €2145, resulting in an ICER of €7083 (Table 3). In this scenario, the relative reduction in clinical event rates also improved. The number of dialysis and kidney transplantation events decreased by 18.5% and 19.0%, respectively, and the incidence of non-fatal MIs, non-fatal strokes, and fatal CVE decreased by 9.7%, 8.0%, and 3.6%, respectively (Appendix p 34). On the other hand, assuming that not all individuals visiting their GP received treatment optimization, increased the ICER to €20,623. These data suggest that treatment optimization after screening further improves the cost-effectiveness of home-based albuminuria screening.

**Fig. 4:**
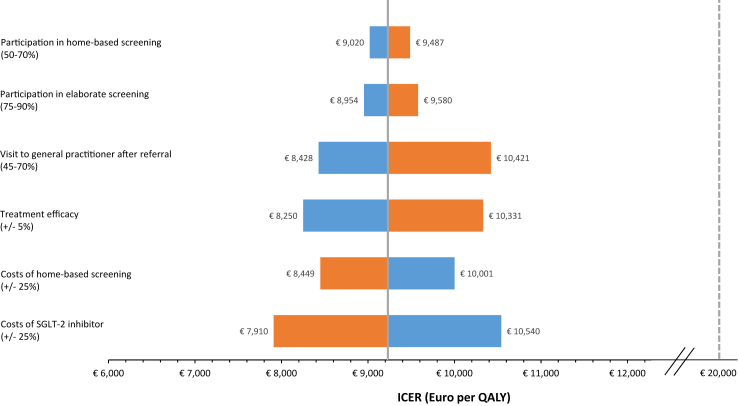
**Sensitivity analyses demonstrating the influence of various model inputs relative to the base-case incremental cost effectiveness ratio (€9225 per QALY)**. The continuous line depicts the ICER derived from the base-case (€9225/QALY). The dotted line represents the €20,000/QALY willingness-to-pay threshold. The blue bars depict the ICER yielded with the highest variable input, the orange bars depict the ICER yielded with the lowest variable input.

In the one-way sensitivity analyses, the results were robust to varying several input parameters and home-based screening remained cost-effective (Fig. 4). The costs of SGLT-2 inhibitor treatment appeared to be the most impactful parameter. The ICER would decrease to €7910 when assuming that SGLT-2 inhibition treatment would be 25% cheaper.

### Subgroup analyses

Compared with the base-case analysis, implementing home-based albuminuria screening in the 45–64 years subgroup led to higher incremental QALY gain (0.27) and a lower ICER of €7946/QALY. Conversely, implementing screening in the older subgroup of 65–80 years increased the ICER to €10,310 (Table 3).

### Budget impact analysis

**Fig. 5:**
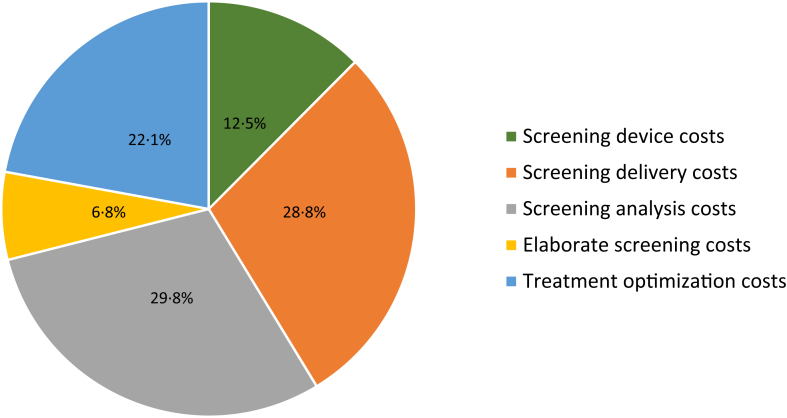
**Distribution of total estimated budget impact of €99.2 million when implementing the one-time home-based screening strategy and initiating treatment for 7,545,845 invited individuals (all 45-80-year-old individuals in the Netherlands in 2021) for albuminuria in the base-case analysis**.

Implementing a one-time home-based albuminuria screening for all individuals aged 45–80 in the Netherlands in 2021 (n = 7,545,845) would lead to an estimated budget impact of €99.2 million over a one-year period (Table 4, Appendix pp 35–36). This would lead to a budget impact of €13 per invited individual and €789 per individual identified with increased albuminuria attending the elaborate screening. The home-based screening, elaborate screening, and treatment optimization contributed 70.1%, 6.8%, and 22.1% to the total budget impact, respectively (Fig. 5). Delivery costs and laboratory analysis costs contributed most to the home-based screening costs. In fact, 55.2% of the budget impact (€54.7 million) was caused by the costs related to delivery and laboratory analysis of the first home-based screening tests.

**Table 4:** One-year budget impact of the home-based albuminuria screening using a UCD versus usual care, in case all 45–80 year old individuals in the Netherlands in 2021 were screened (n = 7,545,845), based on costs, participation rates and yield of screening in THOMAS.

	Individuals usual care (n)^a^	Individuals screening (n)^a^	Costs per individual^b^	Total costs usual care^a^	Total costs screening^a^	BI screening vs. usual care^a^
**Cost item of home-based albuminuria screening**						
**Home-based screening**						
Screening device + delivery to individual	–	7,545,845	€3.82	–	€28,825,128	€28,825,128
Delivery screening device to laboratory + analysis in laboratory	–	4,503,327	€8.38	–	€37,737,878	€37,737,878
Confirmatory screening device + delivery to individual	–	238,805	€3.82	–	€912,236	€912,236
Delivery confirmatory screening device to laboratory + analysis in laboratory	–	221,819	€8.38	–	€1,858,844	€1,858,844
Confirmatory screening device 2 + delivery to individual	–	99,918	€3.82	–	€381,689	€381,689
Delivery confirmatory screening device 2 to laboratory + analysis in laboratory	–	92,695	€8.38	–	€776,788	€776,788
**Elaborate screening**						
Elaborate screening of positive individuals	–	125,760	€53.8	–	€6,765,883	€6,765,883
**Treatment optimization**						
Visiting general practitioner	–	66,843	€35.7	–	€2,385,775	€2,385,775
Starting or continuing treatment	54,953	98,820	€442^c^	€24,268,764	€43,836,625	€19,567,861
**Total BI**	–	–	–	–	–	€99,212,082

BI = budget impact. GP=General Practitioner. N = number.

a Rounded and derived from the probabilities to participate in THOMAS as shown in Table S2 of the Appendix.

b Costs of the individual cost items are as follows: €1.57 for screening device; €2.25 for delivery to individual and delivery to laboratory; €6.13 for analysis in laboratory.

c Rounded weighted average of yearly treatment costs using both strategies.

## Discussion

We developed a HE model, informed by individual-level data from the THOMAS study, to estimate the potential clinical and economic effects of home-based albuminuria screening among the general population aged 45–80 from the Dutch healthcare perspective. We found that home-based albuminuria screening is likely cost-effective with an ICER of €9225/QALY. Further optimizing treatment optimization by treating all screening individuals with newly diagnosed risk factors or risk factors not within target values improved the ICER to €7083/QALY. Limiting screening to those aged 45–65 appeared more cost-effective than limiting screening to those aged 65–80. The one-year budget impact was estimated at €99.2 million for approximately 7.5 million individuals to be invited for the screening.

Our base-case analysis suggests that home-based albuminuria screening of the general population is cost-effective and led to more health benefits compared to usual care, with an ICER of €9225/QALY, derived from the incremental costs of €1607 and incremental QALY gain of 0.17. Moreover, we showed that screening prevented clinical events compared to usual care. The life expectancy of an average Dutch individual aged 70 (the average age of individuals in our synthetic cohort) is 16 years, compared to the average life expectancy for individuals participating in the screening is calculated to be 8.95 for usual care and 9.14 for the screening strategy.^41^ Importantly, the difference in life expectancy between an average Dutch individual and those participating in the screening reflects that individuals identified in the screening have albuminuria and related co-morbidities, whereas a large part of the general population does not. The QALY and LY gain may not appear to be large. This is due to the fact that it reflects the average benefit across all individuals who were screened, while only those with undetected disease may benefit from screening. It is, therefore, better to compare the efficacy of our screening strategy to screening strategies evaluated in other medical domains, including cardiovascular disease and cancer screening.^42^, ^43^, ^44^ In this way, the QALY gain by our screening is relatively large. For instance, the QALY gain with cardiovascular risk screening among patients with rheumatoid arthritis is 0.09 per screened individual.^43^ Although the number of prevented events may not be as high as expected, life expectancy is also prolonged by screening and initiating preventive treatment, allowing more time for events to occur. Importantly, the estimated ICER is well below commonly applied willingness-to-pay thresholds, which vary widely per country. For example, in the United States and the United Kingdom, respectively, general thresholds of $100,000–$150,000 and £20,000–£30,000 are applied, whereas in the Netherlands, a specific threshold of €20,000 is accepted for implementing preventive programs.^39^^,^^45^^,^^46^ Of note, home-based screening had a high probability of being cost-effective according to this relatively strict Dutch threshold of €20,000. Our findings are in contrast to most previous cost-effectiveness studies performed in the field of CKD, which reported that CKD screening (either albuminuria/proteinuria-based screening or eGFR-based screening) in the general population is not cost-effective.^16^, ^17^, ^18^ A recent systematic review by Yeo et al. showed widely varying ICERS ranging from $661 to $430,595/QALY, as reported by studies investigating the cost-effectiveness of CKD screening.^47^ Our study differed in two important aspects from these studies, which could explain the difference in cost-effectiveness: the methodology of the applied models and the screening strategy.

With regard to the applied models, most previous studies only considered savings and health gains related to progression of kidney disease.^16^^,^^17^^,^^48^ When screening the general population, most patients with moderately increased albuminuria or mildly decreased kidney function are unlikely to experience kidney failure. Instead, many will experience a CVE, highlighting the importance of initiating preventive treatments such as blood pressure control and RAS inhibitors that confer both kidney- and cardioprotection.^49^

Regarding the chosen screening strategy, there are several differences. First, several previous studies often targeted only severely increased albuminuria, severely reduced eGFR, or a combination.^16^^,^^17^ Yet, excluding moderately increased albuminuria or an eGFR between 45 and 60 ml/min/1.73 m^2^ identifies fewer individuals, limits preventive options, and more often requires costly treatment, due to the substantially higher risks for kidney outcomes associated with severely increased albuminuria or an eGFR <45, compared to moderately increased albuminuria or an eGFR 45–60. Second, most previous studies analyzed screening strategies as performed by a primary care physician, of which costs ranged between $50 and 200.^16^, ^17^, ^18^, ^19^ Compared to a home-based screening setting, primary care-based screening would further increase costs and, thus, the ICER. Two prior cost-effectiveness studies with similar home-based (pre-) screening of the general population found cost-effective outcomes in line with our results.^29^^,^^50^ This indicates that home-based screening, rather than primary care-based screening, could enhance the cost-effectiveness of CKD screening. Lastly, the recent systematic review by Yeo et al. emphasized that while treatment effectiveness was identified as one of the most influential factors, most analyses only modelled the preventive effect of RAS inhibition.^47^ Our analysis also incorporated the beneficial effects of blood pressure reduction and recent CKD treatment improvements, including the effect of SGLT2 inhibitors that have shown their kidney- and cardioprotective effect in addition to RAS inhibition. Cusick et al. recently published a cost-effectiveness study in which they modelled albuminuria screening of the general population at the age of 55 by a primary care physician. They showed that when adding SGLT2 inhibition to conventional treatment, albuminuria screening of the general population at the age of 55 remained cost-effective in the US. These findings support this choice to model for the effects of SGLT2 inhibition.^48^

In the subgroup analyses, screening only younger individuals (aged 45 to <65) compared to older individuals (aged 65 to 80) was slightly more cost-effective (ICER of €7946 and €10,310/QALY, respectively) and led to a higher QALY gain (incremental QALY of 0.27 vs. to 0.15). This suggests that earlier identification and intervention is of greater benefit for younger people with, on average, more life years remaining. In contrast, two studies based on data from the PREVEND observational cohort, which included individuals from 28 years on, showed improved cost-effectiveness of albuminuria screening by screening above 50 and 60 years instead from younger age.^29^^,^^50^ This improvement could have been caused by the much lower albuminuria prevalence in the younger population below 50 years. However, Boulware and colleagues also observed improved cost-effectiveness of proteinuria screening by limiting screening to individuals ≥60 years.^16^ The ICER decreased from $282,818 in the base-case (annual screening from age 50) to $53,372/QALY (annual screening from age 60). This less favorable cost-effectiveness in individuals aged >50 compared to individuals aged >60 may be explained by the possibility that the frequency of screening was too high. It can also be attributed to the fact that the authors only took benefits with respect to CKD progression into account, and not cardiovascular benefits.

We conducted several scenario analyses to assess model assumptions and the robustness of the base-case analysis. More conservative treatment adherence rates and assuming full treatment efficacy for individuals not within target values only slightly increased the ICER. Moreover, the cost-effectiveness of home-based albuminuria screening was robust to changing the time to opportunistic diagnosis of risk factors. We also assessed the impact of optimizing our screening strategy. In THOMAS, we found that approximately 55% of those referred to their GP for treatment actually visited their GP.^21^ In the present analysis, if we assumed that only 66% of those individuals visiting their GP received treatment optimization, the ICER increased to €20,623. If all individuals with newly diagnosed or risk factors outside target range would visit their GP upon referral and received adequate treatment, the ICER would improve to €7083/QALY, and more QALYs were gained (0.30 vs. 0.17 in the base-case analysis). Similarly, the reduction in kidney and CVE events improved, compared to the base-case analysis. This underlines the importance of improving CKD awareness among the general population for optimal population-level impact of a screening program, considering that at the moment, the perceived risk of CKD by individuals is generally low.^51^

In the one-year budget impact analysis, we showed that implementing a one-time home-based albuminuria screening for 7.5 million individuals aged 45–80 would lead to a budget impact of €99.2 million in the first year. A one-year analysis of the PREVEND observational cohort study showed a similar budget impact of a one-time albuminuria screening, estimated at €17.6 million per 1 million individuals.^29^ The larger part of costs related to the screening program was induced by the costs of the home-based screening (70%) and only a small part by subsequent treatment (22%). Most home-based screening costs were attributed to the shipment and laboratory analysis of the screening tests. In the base-case analysis with a lifetime horizon, we also identified screening costs as the main drivers of the incremental costs. Efforts to lower these costs could decrease the budget impact and improve cost-effectiveness considerably. For example, in countries where home-based colorectal cancer screening is implemented, lowering costs could be achieved by combining home-based screening for albuminuria with such a screening, because this would lower the costs for the postal process. Similarly, the cost-effectiveness will be improved by the likely decrease in the costs of SGLT2-inhibitor as patents will expire.^52^ To our knowledge, this is the first cost-effectiveness analysis based on prospective data of a real-world implementation study for home-based albuminuria screening rather than using data from surveys, cohort studies, or randomized clinical trials.^21^ Several important questions remain for implementation of albuminuria screening can be considered. Investigating the effect of repeat screening and optimal time interval between screenings is necessary to estimate the impact on cost-effectiveness. We examined a single albuminuria screening without repeated screening, while prior evidence suggests that the cost-effectiveness of CKD screening may improve with increased screening intervals.^47^ Future studies could also explore whether the testing interval and frequency of screening could be personalized based on individual previous risk status, as determined among others by albuminuria and kidney function values. Moreover, our study's context was a high-income country. CKD prevalence is higher in low- and middle-income countries compared to high-income countries. As described by Okpechi et al., the availability of policies for guidance on early CKD identification is especially low in low-income countries.^53^ It is, therefore, plausible that home-based albuminuria screening could also be cost-effective in low-income countries. On the other hand, especially for those countries, it has been argued that investing costs and resources in the early detection of CKD would result in limited funds available for managing known individuals with CKD.^54^ Variations in healthcare resources and costs between countries require that the cost-effectiveness is further investigated, tailored to a country's CKD and albuminuria prevalence, resources, and income.

Several limitations should be mentioned. First, we could only include gains related to early albuminuria identification impacting the occurrence of kidney and CVEs. We were not able to include the possibility of experiencing CVEs after a kidney event and gains related to newly diagnosed or treatment optimization for diabetes type 2 and newly diagnosed heart failure. We estimate that including diabetes type 2 in our analysis would have led to limited benefit as relatively low numbers of newly diagnosed patients with diabetes type 2 were found. However, gains with respect to prevention of other diseases, of which the progression and even development are associated with albuminuria, such as diabetes and heart failure, could further improve the cost-effectiveness of albuminuria population screening if such individuals are found.^55^^,^^56^ Second, due to the absence of follow-up in the THOMAS study, we used the most recently available literature and prediction models to estimate the probabilities of kidney progression, experiencing CVEs, and utilities. The estimation of albuminuria and eGFR progression probabilities was based on data from the observational PREVEND cohort, in which part of the individuals received blood pressure control and RAAS-inhibition treatment after identification.^57^ This may have led to underestimating the progression rate in our model. Furthermore, extrapolating treatment effects derived from clinical trials may not completely capture the situation in the general CKD population. Third, our analyses were performed from a healthcare perspective rather than a societal one, as advised by the Dutch guidelines for health economic evaluations. Lastly, the synthetic cohort used for our analyses was derived from a limited number of observations. Future research should aim to include the societal impact of screening on QALYs and nonmedical costs from a societal perspective, and corroborate the findings in prospective and larger datasets.

Our study also has several strengths. We utilized real-world population-based data for our cost-effectiveness analysis derived from the THOMAS study, capturing a heterogeneous, contemporary Dutch population representative of those who would participate in general population screening for albuminuria rather than using survey data or population data from randomized clinical trials. Moreover, we used real-world long-term observational data obtained in the PREVEND study, which together allowed the analysis of the natural course of kidney disease. Additionally, including assumptions on therapy non-adherence further approached a real-life setting. We also included the newest developments in kidney research by including the cardio- and kidney protective effect of SGLT2 inhibitors in addition to RAS inhibition, and by adding albuminuria and eGFR to the SCORE2 algorithm to improve cardiovascular disease risk prediction.^30^^,^^58^

One important point to consider for the present study is that the primary aim is the early identification of CKD. Consequently, not all individuals with CV risk factors will be identified, because many will have normal albuminuria. However, previous studies have shown that individuals without albuminuria but newly discovered CV risk factors have, on average, an absolute risk of CV disease that does not merit start of cardioprotective treatment.^59^ On the contrary, in individuals with increased albuminuria and newly discovered risk factors, the start of cardioprotective treatment is indicated due to the high average CV risk. In addition, a recent report from the World Health Organization showed that current evidence indicates that systematic population-level screening for CV risk factors, such as screening for hypertension, is not (cost-)effective in reducing the burden of cardiovascular disease.^60^ These considerations justify the focus on albuminuria as starting point for screening in order to improve cardiorenal health in subjects with CKD.

In conclusion, using data from a contemporary sample of the Dutch general population aged 45 to 80, our study suggests that home-based albuminuria screening of the general population is cost-effective below established willingness-to-pay thresholds to prevent kidney and cardiovascular-related outcomes. Our study results offer new insights for the implementation of such albuminuria screening.

## Contributors

LMK, RTG, BE-R, MHMT, MHH, and RWvE conceptualized the study underlying the present manuscript. LMK, RTG, DvM, BE-R, MHMT, and RWvE were involved in the conduct of the study underlying the present manuscript. XGLVP, DvM, LMK, RTG, and HK conceptualized the present manuscript. XGLVP and HK developed the methods, with input from DvM, LMK and RTG. XGLVP performed the formal analysis. DvM, XGLVP, LMK, RTG and HK wrote the original draft of te manuscript. BE-R, MHMT, MHH, MLPL, RWvE, HJLH, and CB, reviewed and edited the manuscript. XGLVP, DvM, LMK, RTG and HK directly accessed and verified the underlying data, and all authors took the responsibility for the decision to submit for publication.

## Data sharing statement

De-identified and anonymized participant data used in the study are available upon reasonable request to the corresponding author, approval by the joint investigators, and with a signed data access agreement.

## Declaration of interests

In the past three years, RTG has received fees for consultancy or grants, or both, for research from AbbVie, AstraZeneca, Baxter, Bayer, Healthy.io, Roche, and Sandoz. HJLH has received fees for lectures, consultancy, and grants for research from Astellas, AstraZeneca, Bayer, Boehringer Ingelheim, Chinook, CSL Pharma, Dimerix, Eli Lilly, Fresenius, Gilead, Janssen, Novo Nordisk, and Travere Therapeutics. CB received fees for lectures, consultancy, and travel support from AstraZeneca, Boehringer Ingelheim, and MundiPharma. CB is scientific advisor for VEROZ and holds stock in Health-Ecore, Digital Health Link, SensUR Health, PITTS, and Pharmecore Holding. MHH received fees for consultancy and grants from Bayer, Vifor, and AstraZeneca. XGLVP received the Comenius Teaching Fellowship grant. All other authors declare no competing interests.
