# Supplementary material for: Cost-effectiveness of home-based screening of the general population for albuminuria to prevent progression of cardiovascular and kidney disease
Source: eClinicalMedicine. 2024 Jan 17;68:102414. doi: 10.1016/j.eclinm.2023.102414 (PMC10827681; doi:10.1016/j.eclinm.2023.102414)
Supplement: Appendix [file mmc1.docx]

Supplement to: Cost-effectiveness of home-based screening of the general population for albuminuria to prevent progression of cardiovascular and kidney disease

X.G.L.V. Pouwels, D. van Mil, L.M. Kieneker, C. Boersma, R.W. van Etten, B. Evers-Roeten, H.J.L. Heerspink, M.H. Hemmelder, M.L.P. Langelaan, M.H.M. Thelen, R.T. Gansevoort, H. Koffijberg

**Table of Contents**

[Introduction 3](#_Toc146189747)

[Methods 4](#_Toc146189748)

[Population 4](#_Toc146189749)

[Modules 5](#_Toc146189750)

[Screening module 5](#_Toc146189751)

[Determine treatment module 6](#_Toc146189752)

[CKD progression module 7](#_Toc146189753)

[Cardiovascular event module 9](#_Toc146189754)

[Background mortality module 10](#_Toc146189755)

[Determine effects and costs module 11](#_Toc146189756)

[Update patient characteristics 12](#_Toc146189757)

[Post-event module 12](#_Toc146189758)

[Model inputs 13](#_Toc146189759)

[Probabilities to participate in the screening process 13](#_Toc146189760)

[Albuminuria progression 14](#_Toc146189761)

[Yearly eGFR decline per albuminuria category 16](#_Toc146189762)

[Determining probability of cardiovascular event per individual 17](#_Toc146189763)

[Distribution of different types of events by age category 19](#_Toc146189764)

[Treatment effectiveness 20](#_Toc146189765)

[Hazard ratio for death based on albuminuria and eGFR categories 21](#_Toc146189766)

[General population mortality probabilities 22](#_Toc146189767)

[Utility values 23](#_Toc146189768)

[Costs 24](#_Toc146189769)

[Probabilistic analysis details - CVE risk distribution 26](#_Toc146189770)

[Model validation 27](#_Toc146189771)

[AdViSHE 27](#_Toc146189772)

[TECH-VER 27](#_Toc146189773)

[Results 28](#_Toc146189774)

[Validation 28](#_Toc146189775)

[AdViSHE 28](#_Toc146189776)

[TECH-VER 31](#_Toc146189777)

[Events over time 33](#_Toc146189778)

[Event rates and lifetime probability of kidney and cardiovascular events for scenario analysis 5 34](#_Toc146189779)

[One-year budget impact analysis home-based screening versus usual care – screening all 45-80 year old individuals in the Netherlands in 2021 (n = 7,545,845) 35](#_Toc146189780)

[R session information 37](#_Toc146189781)

[References 38](#_Toc146189782)

# Introduction

This document starts with an extensive explanation of model assumptions and structure. The model inputs are then provided. Validation efforts are described and supplementary results are provided.

# Methods

The cost effectiveness of the home-based screening versus usual care was assessed using a patient-level health state transition model, also called microsimulation. All analyses were performed using the statistical software R, version 4.0.3.

## Population

Individuals included in the current health economic analysis fulfilled the inclusion criteria of the THOMAS study. ^1^ The study is registered with ClinicalTrials.gov, NCT04295889.

To populate the model, a synthetic cohort of 100,000 individuals was generated using the individual-level data from the THOMAS study and the synthpop R package. ^1,2^ Baseline characteristics of the synthetic cohort and a comparison with the original data are provided in the manuscript.

## Modules

This section provides an overview of the different modules that were defined in the health economic model.

### Screening module

This module determines the number of individuals who undergo the different phases of screening and determines the number of individuals who will enter the simulation. The results of this module are also used to determine the cost of the screening process in the home-based screening.

**Figure S1. Screening module.**FU=follow-up test (confirmatory test).


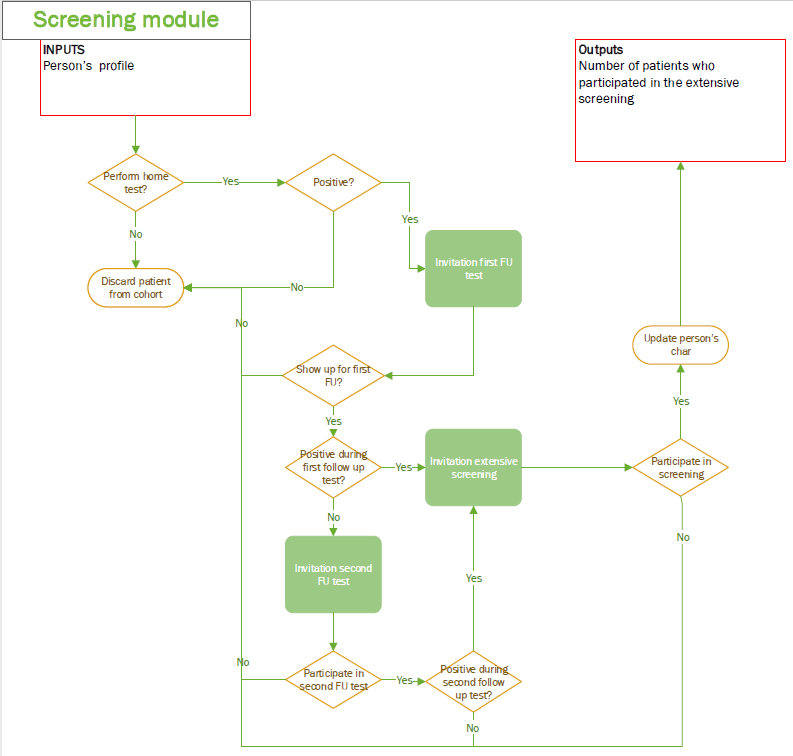


### Determine treatment module

This module determines whether an individual (starts or) continues the treatment that he/she receives. In the base-case analysis, all newly diagnosed individuals or individuals with poorly controlled risk factors are assumed to start treatment if they go to the GP as recommended during the extensive screening. The already diagnosed individuals are assumed to continue treatment.

**Figure S2. Determine treatment module.**

GP=general practitioner.


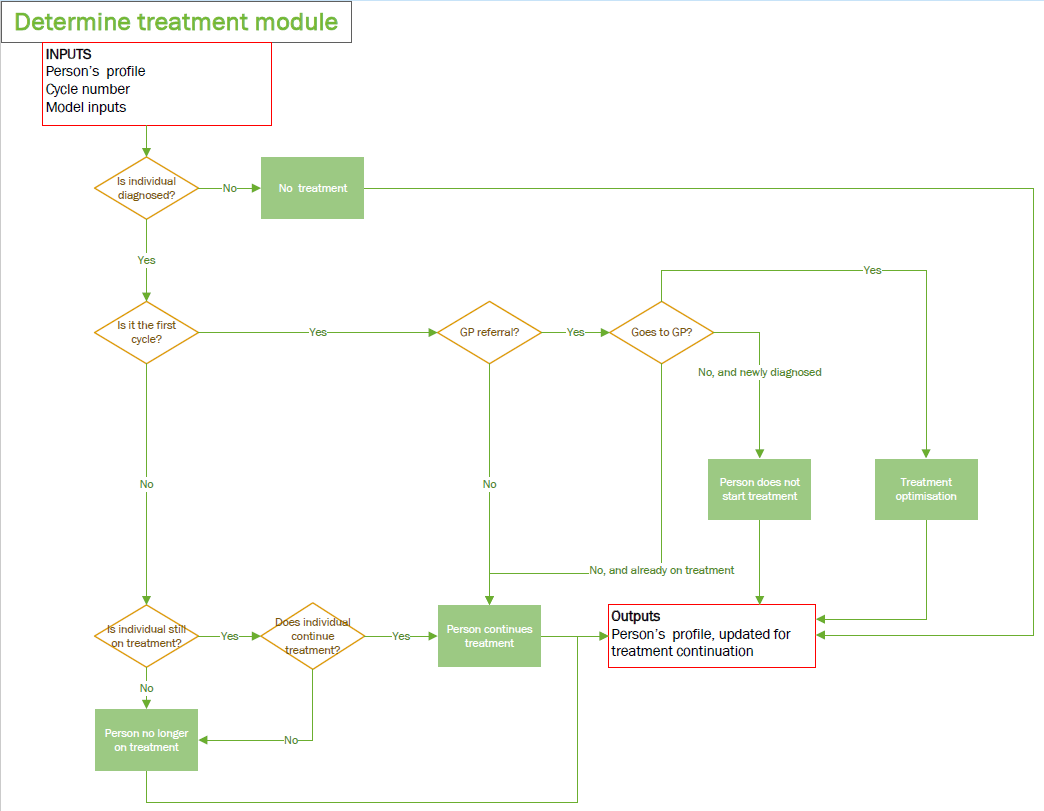


### CKD progression module

This module determines the albuminuria level, eGFR, and CKD class of individuals, based on whether or not they receive treatment. The CKD risk classes in this model are based on the combination of albuminuria level and eGFR as defined by the KDIGO 2012 Clinical Practice Guideline and the Dutch General Practitioner Society’s guidelines. ^3^ These risk classes are used to assign health utility values and costs to each individuals. Hence, the original risk classification has slightly been modified to differentiate between the eGFR levels 3a, 3b, 4, and 5 (these last two being the same class in the KDIGO classification but not in this model). During each cycle, an individual can transit between different albuminuria level, which determines the yearly eGFR decrease of that individual. Albuminuria progression or regression is based on the paper of Brantsma and colleagues. ^4^ In the current analysis, albumin-to-creatinine ratio (ACR) of individuals, which was determined during the extensive screening. The baseline eGFR was also determined during the extensive screening. The Figure below displays the CKD progression module and the table shows how ACR was converted to 24-hour urinary albumin excretion (UAE) used by Brantsma and colleagues in the current health economic model. ^4^

**Figure S3. CKD progression module.**

CKD=chronic kidney disease. GFR, glomerular filtration rate. UAE=24-hour urinary albumin excretion.


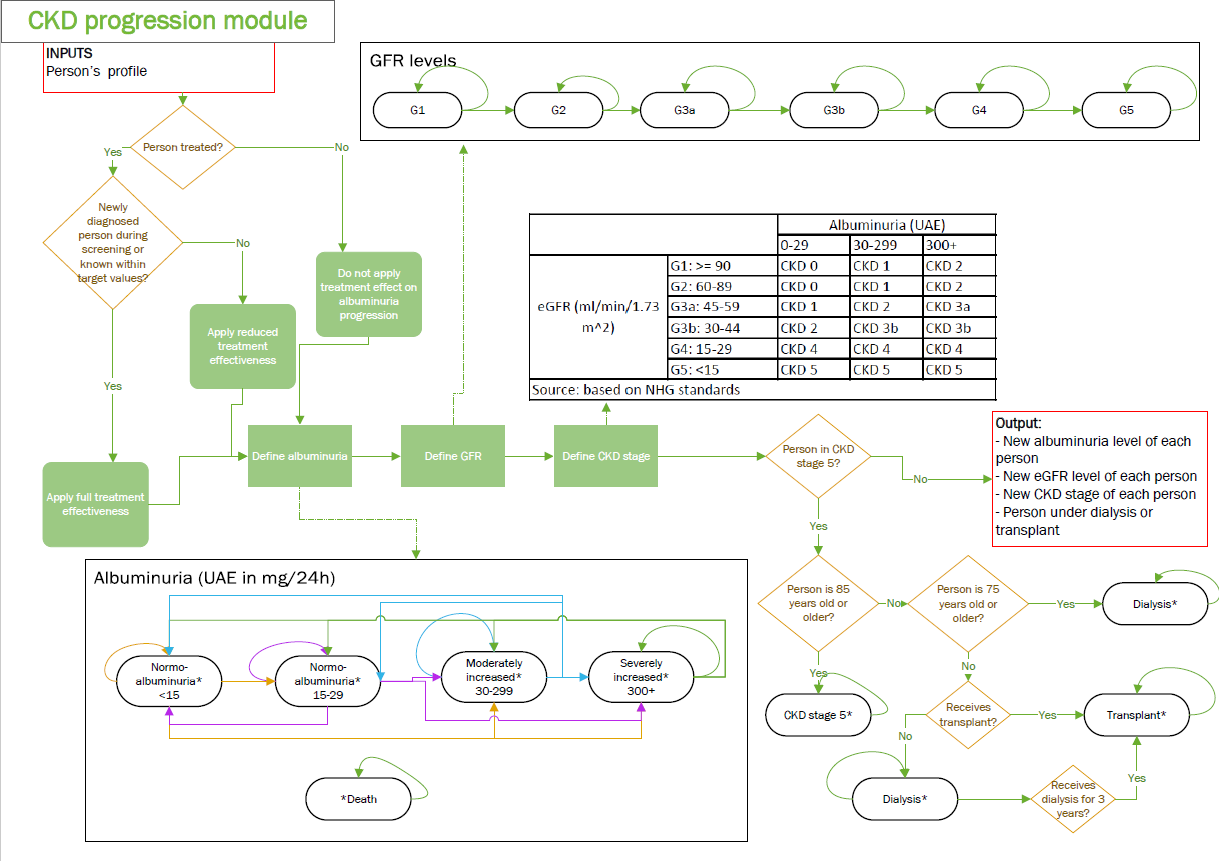


**Table S1. Conversion ACR - UAE used in the current model.**

| **ACR (mg/mmol)** | **UAE (mg/24h)** | **Albuminuria category** |
| --- | --- | --- |
| < 1·5 | <15 | Normal low |
| 1·5-2·99 | 15 - 29·99 | Normal high |
| 3·0 - 29·99 | 30 - 299·99 | Moderately-increased albuminuria |
| ≥ 30 | ≥ 300 | Severely-increased albuminuria |

UAE=24-hour urinary albumin excretion. ACR=Albumin-to-creatinine ratio

### Cardiovascular event module

In the Cardiovascular events module, it is determined whether individuals experience a cardiovascular event (CVE), and which type of CVE occurs. The CVE included in the current health economic model are: non-fatal myocardial infarction, non-fatal stroke and fatal cardiovascular events as defined in the SCORE2 prediction model (ICD 10 codes). SCORE2 and its add-on for albuminuria and eGFR levels were used to determine the probability of experiencing a CVE. ^5^

**Figure S4. Cardiovascular event module.**CVD=cardiovascular disease. MI = myocardial infarction.


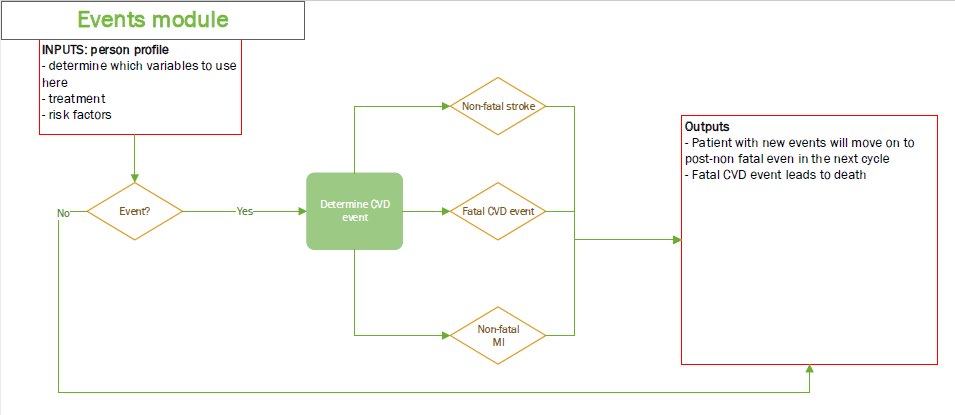


### Background mortality module

Background mortality refers to causes of death other than CKD- and CVE-related death. This background mortality is based on the Dutch general population mortality probabilities provided by the Dutch National Statistics (CBS 2021). ^6^ To prevent double-counting with death related to CKD and CVEs, the ICD10 codes relating to CKD (N18) and to the CVEs included in the SCORE2 prediction model (ICD10: I10-I16, I20-I25, I46-I52, I60-I69, I70-I73, R96, excluding I60 and I62) were removed when determining these probabilities.

**Figure S5. Background mortality module.**eGFR=estimated glomerular filtration rate.

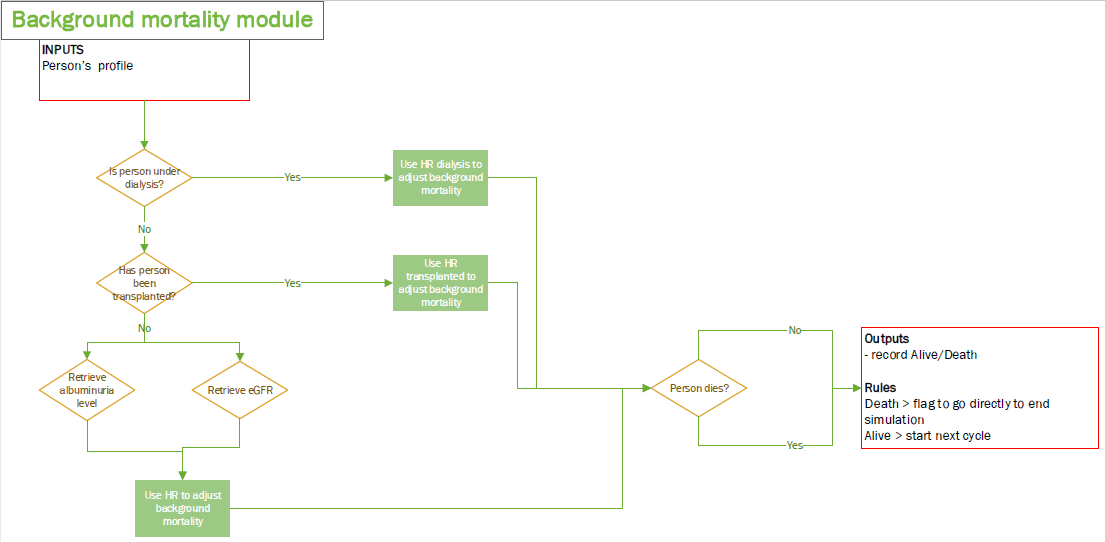


### Determine effects and costs module

This module determines the effects and costs accrued by each individual in the simulation during each cycle based on their CKD class, and whether they experience a CVE or are under dialysis or transplantation.

**Figure S6. Determine effects and costs module.**QoL=quality of life. CKD=chronic kidney disease.

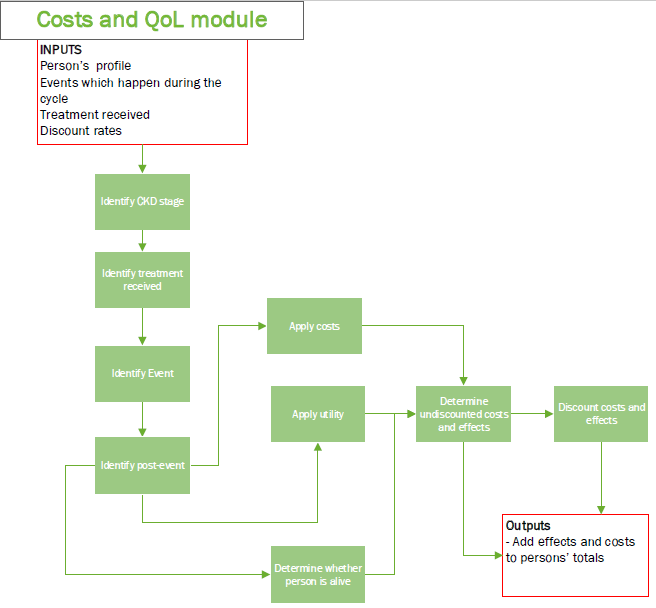


### Update patient characteristics

In this module, the following individuals’ characteristics are updated: age and time since (non-)fatal CVE (if individual experienced a CVE event in previous cycles).

### Post-event module

Individuals who experienced a CVE, are under dialysis, or received a transplant do not move through other modules, hence, they remain in their post-event state. These individuals only accrue costs according to their post-event health state in the Determine effects and costs module.

## Model inputs

Model inputs are presented per category in the following sections.

### Probabilities to participate in the screening process

In the Screening module, the probabilities associated with participating in the home screening (first and confirmatory tests), and the extensive screening were determined for each screening strategy separately, based on the THOMAS study. ^1^ For the subgroup analyses, these probabilities were defined for the subgroups 45-64 and 65-80 years old separately. These probabilities are conditional on participating in the previous step(s). For example, the probabilities of participating the first confirmatory test is only applied to individuals who participated and had a positive initial home test.

**Table S2. Probabilities to participate in the screening process.**

| **Description** | **Mean** | **N event** | **N total** | **Source** | **Distribution** |
| --- | --- | --- | --- | --- | --- |
| Probability to be invited | 100% | 7552 | 7552 | Van Mil et al. ^1^ | Beta |
| Probability to participate in home test | 60% | 4507 | 7552 | Van Mil et al.^1^ | Beta |
| Probability to be positive during home test | 5% | 239 | 4507 | Van Mil et al.^1^ | Beta |
| Probability to participate in first confirmatory test | 93% | 222 | 239 | Van Mil et al. | Beta |
| Probability to be positive during first confirmatory test | 63% | 139 | 222 | Van Mil et al.^1^ | Beta |
| Probability to participate in second confirmatory test | 93% | 77 | 83 | Van Mil et al.^1^ | Beta |
| Probability to be positive during second confirmatory test | 14% | 11 | 77 | Van Mil et al.^1^ | Beta |
| Probability to participate in extensive screening | 83% | 124 | 150 | Van Mil et al.^1^ | Beta |
| Probability to go to the GP after referral | 57% | 54 | 94 | Van Mil et al.^1^ | Beta |
| Probability to be invited (45-64y) | 100% | 4867 | 4867 | Van Mil et al.^1^ | Beta |
| Probability to participate in home test (45-64y) | 57% | 2762 | 4867 | Van Mil et al.^1^ | Beta |
| Probability to be positive during home test (45-64y) | 3% | 72 | 2762 | Van Mil et al.^1^ | Beta |
| Probability to participate in first confirmatory test (45-64y) | 93% | 67 | 72 | Van Mil et al.^1^ | Beta |
| Probability to be positive during first confirmatory test (45-64y) | 60% | 40 | 67 | Van Mil et al.^1^ | Beta |
| Probability to participate in second confirmatory test (45-64y) | 85% | 23 | 27 | Van Mil et al.^1^ | Beta |
| Probability to be positive during second confirmatory test (45-64y) | 13% | 3 | 23 | Van Mil et al.^1^ | Beta |
| Probability to participate in extensive screening (45-64y) | 81% | 35 | 43 | Van Mil et al.^1^ | Beta |
| Probability to be invited (65-85y) | 100% | 2685 | 2685 | Van Mil et al.^1^ | Beta |
| Probability to participate in home test (65-85y) | 65% | 1745 | 2685 | Van Mil et al. ^1^ | Beta |
| Probability to be positive during home test (65-85y) | 10% | 167 | 1745 | Van Mil et al. ^1^ | Beta |
| Probability to participate in first confirmatory test (65-85y) | 93% | 155 | 167 | Van Mil et al. ^1^ | Beta |
| Probability to be positive during first confirmatory test (65-85y) | 64% | 99 | 155 | Van Mil et al. ^1^ | Beta |
| Probability to participate in second confirmatory test (65-85y) | 96% | 54 | 56 | Van Mil et al. ^1^ | Beta |
| Probability to be positive during second confirmatory test (65-85y) | 15% | 8 | 54 | Van Mil et al. ^1^ | Beta |
| Probability to participate in extensive screening (65-85y) | 83% | 89 | 107 | Van Mil et al. ^1^ | Beta |

N=number. GP=general practitioner. Y=years.

### Albuminuria progression

The following table describes the yearly transition probabilities between the different albuminuria levels, based on the work from Brantsma and colleagues, when an individual does not receive any treatment. ^4^

**Table S3. Transition probabilities between albuminuria levels.**

| **Description** | **mean** | **SD** | **n_pt** | **Source** | **Distribution** |
| --- | --- | --- | --- | --- | --- |
| Transition probability between albuminuria levels (NL-NH) | 0·014 | 0·002 | 5007 | Brantsma et al.^4^ | DIRICHLET |
| Transition probability between albuminuria levels (NL-Mod_inc) | 0·003 | 0·001 | 5007 | Brantsma et al. ^4^ | DIRICHLET |
| Transition probability between albuminuria levels (NL-Sev_inc) | <0·001 | 0·000 | 5007 | Brantsma et al. ^4^ | DIRICHLET |
| Transition probability between albuminuria levels (NL-D) | 0·003 | 0·001 | 5007 | Brantsma et al. ^4^ | DIRICHLET |
| Transition probability between albuminuria levels (NL-remain) | 0·98 | 0·002 | 5007 | Brantsma et al. ^4^ | DIRICHLET |
| Transition probability between albuminuria levels (NH-NL) | 0·139 | 0·009 | 965 | Brantsma et al. ^4^ | DIRICHLET |
| Transition probability between albuminuria levels (NH-Mod_inc) | 0·05 | 0·006 | 965 | Brantsma et al. ^4^ | DIRICHLET |
| Transition probability between albuminuria levels (NH-Sev_inc) | <0·001 | 0·027 | 965 | Brantsma et al. ^4^ | DIRICHLET |
| Transition probability between albuminuria levels (NH-D) | 0·007 | 0·017 | 965 | Brantsma et al. ^4^ | DIRICHLET |
| Transition probability between albuminuria levels (NH-remain) | 0·803 | 0·032 | 965 | Brantsma et al. ^4^ | DIRICHLET |
| Transition probability between albuminuria levels (Sev_inc-NL) | 0·006 | 0·006 | 80 | Brantsma et al. ^4^ | DIRICHLET |
| Transition probability between albuminuria levels (Sev_inc-NH) | 0·003 | 0·008 | 80 | Brantsma et al. ^4^ | DIRICHLET |
| Transition probability between albuminuria levels (Sev_inc-Mod_inc) | 0·063 | 0·003 | 80 | Brantsma et al. ^4^ | DIRICHLET |
| Transition probability between albuminuria levels (Sev_inc-D) | 0·023 | 0·004 | 80 | Brantsma et al. ^4^ | DIRICHLET |
| Transition probability between albuminuria levels (Sev_inc-remain) | 0·905 | 0·011 | 80 | Brantsma et al. ^4^ | DIRICHLET |
| Transition probability between albuminuria levels (Mod_inc-NL) | 0·036 | 0·011 | 812 | Brantsma et al. ^4^ | DIRICHLET |
| Transition probability between albuminuria levels (Mod_inc-NH) | 0·049 | 0·007 | 812 | Brantsma et al. ^4^ | DIRICHLET |
| Transition probability between albuminuria levels (Mod_inc-Sev_inc) | 0·009 | 0·001 | 812 | Brantsma et al. ^4^ | DIRICHLET |
| Transition probability between albuminuria levels (Mod_inc-D) | 0·016 | 0·003 | 812 | Brantsma et al. ^4^ | DIRICHLET |
| Transition probability between albuminuria levels (Mod_inc-remain) | 0·89 | 0·013 | 812 | Brantsma et al. ^4^ | DIRICHLET |

D=Death. n_pt=number of observation on which estimate is based. NL=Normal low. NH=Normal high. Mod_inc=Moderately-increased albuminuria. SD=standard deviation. Sev_inc=Severely-increased albuminuria.

In the HE model, the above-described transition probabilities were adjusted since they were obtained from a population in which younger individuals were included than the current HE model. The rates by which these transition probabilities were adjusted are provided in the table below and were obtained by taking the ratio of the *Age ≥ 50 Years* column by the *All subjects* column from Appendix II of Boersma and colleagues. ^7^ These rates were applied using the probability-rate and rate-probability conversion calculations from Fleurence and Hollenbeak. ^8^

**Table S4. Adjustment transition probabilities.**

| **Transition probability** | **Rate used for adjustment** |
| --- | --- |
| NL - NH | 1·312 |
| NL - Mod_inc | 1·241 |
| NL - Sev_inc | 0·221 |
| NH - Mod_inc | 1·15 |
| NH - Sev_inc | 0·297 |
| NH - NL | 0·74 |
| Mod_inc - Sev_inc | 13·962 |
| Mod_inc - NH | 0·545 |
| Mod_inc - NL | 0·714 |
| Sev_inc - Mod_inc | 1·067 |
| Mod_inc - NH | 0·604 |
| Mod_inc - NL | 0·765 |

NL=Normal low. NH=Normal high.Mod_inc=Moderately-increased albuminuria. Sev_inc=Severely-increased albuminuria.

### Yearly eGFR decline per albuminuria category

The following table describes the yearly eGFR decline (in ml/min/1.73m^2^) per albuminuria and age (until 55, and above 55 years old) categories, based on data from van der Velde and colleagues. ^9^ In the health economic model, the UAE categories 30-149.99 and 150-299.99 are combined within the moderately-elevated albuminuria category. The eGFR annual decline from this category is calculated based on a weighted average of the categories 30-149.99 and 150-299.99 mg/24h. This weighted average was based on the number of patients in each of these two categories as reported by van der Velde and colleagues (weight accorded to the 30-149.99 category = 178/(178+77) = 0.7). ^9^

**Table S5. Yearly eGFR decline.**

| **Description** | **mean** | **SD** | **n_pt** | **Source** | **Distribution** |
| --- | --- | --- | --- | --- | --- |
| Annual eGFR decline for UAE category 0-15 mg/24h - ≤ 55 years | 0·33 | 2·09 | 3767 | Van der Velde et al.^9^ | GAMMA |
| Annual eGFR decline for UAE category 15-29.99 mg/24h - ≤55 years | 0·39 | 1·62 | 620 | Van der Velde et al.^9^ | GAMMA |
| Annual eGFR decline for UAE category 30-149.99 mg/24h - ≤ 55 years | 0·41 | 1·59 | 375 | Van der Velde et al.^9^ | GAMMA |
| Annual eGFR decline for UAE category 150-299.99 mg/24h - ≤ 55 years | 0·58 | 1·57 | 34 | Van der Velde et al.^9^ | GAMMA |
| Annual eGFR decline for UAE category 300+ mg/24h - ≤ 55 years | 1·11 | 1·57 | 34 | Van der Velde et al.^9^ | GAMMA |
| Annual eGFR decline for UAE category 0-15 mg/24h - > 55 years | 0·26 | 4·44 | 1223 | Van der Velde et al.^9^ | GAMMA |
| Annual eGFR decline for UAE category 15-29.99 mg/24h - > 55 years | 0·59 | 2·89 | 357 | Van der Velde et al.^9^ | GAMMA |
| Annual eGFR decline for UAE category 30-149.99 mg/24h - > 55 years | 0·62 | 2·97 | 373 | Van der Velde et al.^9^ | GAMMA |
| Annual eGFR decline for UAE category 150-299.99 mg/24h - > 55 years | 1·18 | 1·95 | 43 | Van der Velde et al.^9^ | GAMMA |
| Annual eGFR decline for UAE category 300+ mg/24h - > 55 years | 1·85 | 2·01 | 53 | Van der Velde et al.^9^ | GAMMA |

eGFR=estimated glomerular filtration rate. n_pt=number of observation on which estimate is based. UAE= 24-hour Urinary Albumin Excretion. SD=standard deviation.

### Determining probability of cardiovascular event per individual

The probability of experiencing a CVE was determined for each individual based on their characteristics, the SCORE2 prediction model, and the SCORE2 albuminuria and eGFR add-on ^5^. Since the SCORE2 only predicts the probability of CVE for a period of 10 years and only for individuals between 40-69 years old, the following steps (and assumptions) were taken to estimate the probabilities of experiencing a CVE over the multiple decades. This approach is to the approach described by Lagerweij and colleagues. ^10^ The probability of CVE was assumed to remain constant for individuals aged 80 and more.

1. SCORE2 and the SCORE2 albuminuria and eGFR add-on was applied to individuals aged between 45-69 years old to estimate their 10-year CVE risk; The European low risk region was applied. ^5^

2. CVE risk estimates of individuals in the decades 40-49, 50-59, and 60-69 were converted to SCORE2 estimates for the age 40, 50, and 60, using the same method as Lagerweij and colleagues. ^10^ This conversion provided three risk distributions that were used in the health economic model. This conversion was based on the relative change in the parameters of the distribution fitted to the individuals in the decades 40-49, 50-59, and 60-69. This relative change (per decade), was calculated based on the parameters of the three distribution and the difference in the average age between individuals included in the age categories 40-49, 50-59, and 60-69;

3. Beta distributions were fitted to the three risk distributions (at 40, 50, and 60 years old) to obtain the alpha and beta’s of these beta distributions, which represented the risk distributions for the three decades 40-49, 50-59, and 60-69 years old. To obtain the alphas and betas for the beta distribution for the decades 70-79 years and 80 years (and more), the relative increase in the probability of being hospitalized for or die from a stroke and myocardial infarction (ICD10 numbers used in the SCORE2 calculation) between the age categories 65-80 years and 80+ years was used ^6^. Beta distributions were fitted to these two probability distributions, and the relative increase in alpha and beta was calculated. This relative increase was used to calculate the alpha and beta for the CVE risk distribution of the decade 70-79 and 80+;

4. The place (percentile) of each individual belonging to one of these decade (40-49, 50-59, and 60-69) within the distribution fitted under step 2 was determined. Persons above 69 years old were assigned to a random percentile in these distribution. The percentile assigned to individuals remained the same over the different decades. This means that individuals with a high risk of experiencing a CVE during the extensive screening remained at a high risk of experiencing a CVE during the remaining of their lifetime (and vice versa).

5. The probability of experiencing an event over the different decades per individual was calculated based on the distribution fitted to the different decades and the percentile of each individual within these distributions.

6. The 10-year probability of experiencing a CVE were converted to a yearly probability using the formula provided by Fleurence and Hollenbeak. ^8^

The parameters and mean of the risk distributions for each decade are provided in the Tables below.

**Table S6. Parameters of the beta distributions representing the risk of event per age decade per arm.**

| **Description** | **mean** | **SD** | **n_pt** | **Source** | **Distribution** |
| --- | --- | --- | --- | --- | --- |
| Alpha parameters for CVE risk distributions, decade: 40-49y | 2·069 | NA | 100000 | Van Mil et al. CBS, Matsushita et al. ^1,5,6^ | BOOT |
| Alpha parameters for CVE risk distributions, decade: 50-59y | 2·864 | NA | 100000 | Van Mil et al. CBS, Matsushita et al. ^1,5,6^ | BOOT |
| Alpha parameters for CVE risk distributions, decade: 60-69y | 4·886 | NA | 100000 | Van Mil et al. CBS, Matsushita et al. ^1,5,6^ | BOOT |
| Alpha parameters for CVE risk distributions, decade: 70-79y | 5·177 | NA | 100000 | Van Mil et al. CBS, Matsushita et al. ^1,5,6^ | CALCULATED |
| Alpha parameters for CVE risk distributions, decade: 80y+ | 5·486 | NA | 100000 | Van Mil et al. CBS, Matsushita et al. ^1,5,6^ | CALCULATED |
| Beta parameters for CVE risk distributions, decade: 40-49y | 8·452 | NA | 100000 | Van Mil et al. CBS, Matsushita et al. ^1,5,6^ | BOOT |
| Beta parameters for CVE risk distributions, decade: 50-59y | 11·815 | NA | 100000 | Van Mil et al. CBS, Matsushita et al. ^1,5,6^ | BOOT |
| Beta parameters for CVE risk distributions, decade: 60-69y | 19·842 | NA | 100000 | Van Mil et al. CBS, Matsushita et al. ^1,5,6^ | BOOT |
| Beta parameters for CVE risk distributions, decade: 70-79y | 8·496 | NA | 100000 | Van Mil et al. CBS, Matsushita et al. ^1,5,6^ | CALCULATED |
| Beta parameters for CVE risk distributions, decade: 80y+ | 3·638 | NA | 100000 | Van Mil et al. CBS, Matsushita et al. ^1,5,6^ | CALCULATED |
| 10-year relative difference in probability of experiencing a CVE between decades 40-60y | 1·001 | NA | 100000 | Van Mil et al. CBS, Matsushita et al. ^1,5,6^ | FIXED |
| 10-year relative increase in alpha parameter between decades 60-80y | 0·99 |  |  | CBS. ^6^ | FIXED |
| 10-year relative increase in beta parameter between decades 60-80y | 1·09 |  |  | CBS. ^6^ | FIXED |

CVE=cardiovascular event. n_pt=number of observation on which estimate is based. SD=standard deviation. CBS=Dutch National Statistics. y=years.

**Table S7. Mean probability of experiencing a cardiovascular event per age decade.**

|  | **Mean 10-year risk** | **SD 10-year risk** |
| --- | --- | --- |
| 40-49 years old | 20% | 11·71 |
| 50-59 years old | 20% | 10·01 |
| 60-69 years old | 20% | 7·85 |
| 70-79 years old | 38% | 12·66 |
| 80+ years old | 60% | 15·39 |

SD=standard deviation.

### Distribution of different types of events by age category

The distribution of the types of CVE is based on the number of hospitalizations for and death from stroke and myocardial infarction (for the ICD numbers used in SCORE2) reported by CBS. ^6^ The distributions per age category are the following.

**Table S8. Average probabilities of experiencing cardiovascular events as derived from the Dutch National Statistics.**

|  | **Age category 20-45y** | **Age category 45-65y** | **Age category 65-80y** | **Age category 80y+** |
| --- | --- | --- | --- | --- |
| Probability nonfatal MI men | 0·57 | 0·58 | 0·39 | 0·21 |
| Probability nonfatal stroke men | 0·34 | 0·31 | 0·39 | 0·31 |
| Probability fatal event men | 0·09 | 0·11 | 0·23 | 0·48 |
| Probability nonfatal MI women | 0·32 | 0·44 | 0·31 | 0·15 |
| Probability nonfatal stroke women | 0·62 | 0·46 | 0·47 | 0·31 |
| Probability fatal event women | 0·07 | 0·10 | 0·22 | 0·54 |

Y=years. MI=Myocardial Infarction.

### Treatment effectiveness

The treatment effectiveness of ACE-inhibitors and combination therapy for heart failure ( ACE-inhibitor, betablocker, diuretic) on the transition probabilities between albuminuria categories were based on a previous Dutch health economic evaluation. ^7^ Additionally, ACE-inhibitors (and combination therapy for heart failure patients: ACE-inhibitor, betablocker, diuretic), and statins decreased the probability of experiencing a cardiovascular event. Statins did not have an effect on albuminuria progression, based on work by Baigent and colleagues. ^11^ The relative effectiveness of these treatments on the probability of CKD progression and experiencing a CVE is provided in the table below.

**Table S9. Relative effectiveness of treatments.**

| **Description** | **mean** | **SD** | **Source** | **Distribution** |
| --- | --- | --- | --- | --- |
| Relative risk for the transition from UAE category 15-29·99 to 0-14·99 mg/24h | 1·23 | 1·07 | Boersma et al.^7^ | Lognormal |
| Relative risk for the transition from UAE category 15-29·99 to 30-299·99 mg/24h | 0·74 | 1·16 | Boersma et al.^7^ | Lognormal |
| Relative risk for the transition from UAE category 300+ to 30-299·99 mg/24h | 1·45 | 1·5 | Boersma et al.^7^ | Lognormal |
| Relative risk for the transition from UAE category 30-299·99 to 0-14·99 mg/24h | 1·41 | 1·16 | Boersma et al.^7^ | Lognormal |
| Relative risk for the transition from UAE category 30-299·99 to 15-29·99 mg/24h | 1·16 | 1·18 | Boersma et al.^7^ | Lognormal |
| Relative risk for the transition from UAE category 30-299·99 to 300+ mg/24h | 0·76 | 1·51 | Boersma et al.^7^ | Lognormal |
| Hazard ratio for the occurrence of cardiovascular events when treated with  ACE- inhibitors or combination therapy in case of history with heart failure | 0·71 | 0·60-0·83 | Wei et al.^12^ | Lognormal |
| Relative risk for the occurrence of cardiovascular events when treated with  a statin | 0·75 | 0·70-0·81 | Taylor et al.^13^ | Lognormal |
| Hazard ratio for the occurrence of cardiovascular events and kidney disease progression when receiving SGLT-2 inhibitor | 0·71 | 0·55-0·92 | Heerspink et al.^14^ | Lognormal |
| Relative decrease in treatment effectiveness of treatment in already diagnosed patients | 0·50 | NA | Expert opinion | FIXED |

SD=standard deviation. UAE=24-hour Urinary Albumin Excretion. ACE-inhibitors=angiotensin-converting enzyme inhibitors. SGLT-2 inhibitors=sodium glucose cotransporter-2 inhibitors. NA=not applicable.

### Hazard ratio for death based on albuminuria and eGFR categories

For each combination of albuminuria and eGFR category, the probability of dying from non-renal and non-CVE related causes was adjusted based on hazard ratio’s provided in Matsushita et al. (2010). ^15^ To ensure the estimates from Matsushita et al. (2010) matched the albuminuria categories used in the health economic model, the assumption was made that the urine albumin-to-creatinine ratio (ACR) < 10 = UAE < 15 (Normal low - NL), ACR 10-29 = UAE 15 - 30 (Normal high - NH), ACR 30-299 = UAE 30 - 299 (Moderately increased albuminuria - MIA), and ACR 300+ = UAE 300+ (Severely increased albuminuria - SIA). The hazard ratio’s applied to individuals who are under dialysis and received a kidney transplant were retrieved from Neovius and colleagues. ^16^

**Table S10. Hazard ratios for probability of death due to other causes, per eGFR and albuminuria category.**

| **Description** | **mean** | **95% CI** | **Source** | **Distribution** |
| --- | --- | --- | --- | --- |
| eGFR>100-NL | 1·14 | 1·02-1·27 | Matsushita et al.^15^ | Lognormal |
| eGFR 90-104-NL | 1·00 | 1-1 | Matsushita et al.^15^ | Lognormal |
| eGFR 75-89-NL | 1·00 | 0·91-1·09 | Matsushita et al.^15^ | Lognormal |
| eGFR 60-74-NL | 1·02 | 0·92-1·15 | Matsushita et al.^15^ | Lognormal |
| eGFR 45-59-NL | 1·28 | 1·05-1·57 | Matsushita et al.^15^ | Lognormal |
| eGFR 30-44-NL | 1·97 | 1·59-2·43 | Matsushita et al.^15^ | Lognormal |
| eGFR 15-29-NL | 5·39 | 3·3-8·8 | Matsushita et al.^15^ | Lognormal |
| eGFR>100-NH | 1·52 | 1·28-1·81 | Matsushita et al.^15^ | Lognormal |
| eGFR 90-104-NH | 1·48 | 1·29-1·69 | Matsushita et al.^15^ | Lognormal |
| eGFR 75-89-NH | 1·40 | 1·26-1·55 | Matsushita et al.^15^ | Lognormal |
| eGFR 60-74-NH | 1·49 | 1·34-1·66 | Matsushita et al.^15^ | Lognormal |
| eGFR 45-59-NH | 1·95 | 1·73-2·2 | Matsushita et al.^15^ | Lognormal |
| eGFR 30-44-NH | 2·65 | 2·19-3·22 | Matsushita et al.^15^ | Lognormal |
| eGFR 15-29-NH | 3·66 | 2·43-5·5 | Matsushita et al.^15^ | Lognormal |
| eGFR>100-MIA | 2·32 | 2-2·7 | Matsushita et al.^15^ | Lognormal |
| eGFR 90-104-MIA | 1·61 | 1·39-1·87 | Matsushita et al.^15^ | Lognormal |
| eGFR 75-89-MIA | 1·78 | 1·58-2·01 | Matsushita et al.^15^ | Lognormal |
| eGFR 60-74-MIA | 1·95 | 1·67-2·27 | Matsushita et al.^15^ | Lognormal |
| eGFR 45-59-MIA | 2·51 | 2·16-2·9 | Matsushita et al.^15^ | Lognormal |
| eGFR 30-44-MIA | 3·66 | 2·91-4·6 | Matsushita et al.^15^ | Lognormal |
| eGFR 15-29-MIA | 4·85 | 3·26-7·21 | Matsushita et al.^15^ | Lognormal |
| eGFR>100-SIA | 5·26 | 2·8-9·85 | Matsushita et al.^15^ | Lognormal |
| eGFR 90-104-SIA | 3·65 | 2·13-6·27 | Matsushita et al.^15^ | Lognormal |
| eGFR 75-89-SIA | 2·50 | 1·89-3·31 | Matsushita et al.^15^ | Lognormal |
| eGFR 60-74-SIA | 3·09 | 2·56-3·72 | Matsushita et al.^15^ | Lognormal |
| eGFR 45-59-SIA | 4·10 | 3·39-4·95 | Matsushita et al.^15^ | Lognormal |
| eGFR 30-44-SIA | 5·08 | 4·2-6·15 | Matsushita et al.^15^ | Lognormal |
| eGFR 15-29-SIA | 6·96 | 5·28-9·19 | Matsushita et al.^15^ | Lognormal |
| Under dialysis | 12·60 | 10·8-14·6 | Neovius et al.^16^ | Lognormal |
| Transplanted | 5·60 | 3·5-8·9 | Neovius et al.^16^ | Lognormal |

95% CI=95% Confidence Interval. eGFR=estimated glomerular filtration rate. NL=normal low. NH=normal high. MIA=moderately increased albuminuria. SIA=severely increased albuminuria.

### General population mortality probabilities

The table below shows the age- and sex-dependent general population mortality probabilities which were derived from the Dutch National Statistics. ^6^ The ICD codes involved in SCORE2 and the one dedicated to CKD were removed from the causes of death used to calculated these mortality probabilities.

**Table S11. Yearly probability of death per age category and sex as derived from the Dutch National Statistics.**

| **Age category** | **Men** | **Women** |
| --- | --- | --- |
| 20-24 | 0·04% | 0·02% |
| 25-29 | 0·04% | 0·02% |
| 30-34 | 0·05% | 0·03% |
| 35-39 | 0·07% | 0·04% |
| 40-44 | 0·09% | 0·07% |
| 45-49 | 0·15% | 0·12% |
| 50-54 | 0·24% | 0·21% |
| 55-59 | 0·42% | 0·36% |
| 60-64 | 0·7% | 0·57% |
| 65-69 | 1·11% | 0·8% |
| 70-74 | 1·81% | 1·29% |
| 75-79 | 3·01% | 2·1% |
| 80-84 | 5·34% | 3·72% |
| 85-89 | 9·64% | 7·27% |
| 90-94 | 16·09% | 13·31% |
| 95-104 | 24·57% | 22·25% |
| 105 | 100% | 100% |

###

### Utility values

Based on work by Manns et al. and Okano et al., the quality of life (QALY) of diagnosed and non-diagnosed individuals with kidney function decline was assumed to be the same. ^17,18^ The utility values associated with each CKD class and (post-)CVE are provided in the Table below. Individuals not suffering from any kidney impairment (CKD class 0 in this model), were assumed to have QALY equal to the general Dutch population. ^19^

**Table 12. Utility values associated with each health state and event.**

| **Description** | **mean** | **SD** | **n_pt** | **Source** | **Distribution** |
| --- | --- | --- | --- | --- | --- |
| Utility value associated with CKD stage 0 | 0·900 | 0·170 | 22 | Gorodetskaya et al. ^20^ | BETA |
| Utility value associated with CKD stage 1 | 0·900 | 0·170 | 22 | Gorodetskaya et al. ^20^ | BETA |
| Utility value associated with CKD stage 2 | 0·900 | 0·170 | 22 | Gorodetskaya et al. ^20^ | BETA |
| Utility value associated with CKD stage 3b | 0·870 | 0·240 | 50 | Gorodetskaya et al. ^20^ | BETA |
| Utility value associated with CKD stage 3a | 0·870 | 0·240 | 50 | Gorodetskaya et al. ^20^ | BETA |
| Utility value associated with CKD stage 4 | 0·850 | 0·230 | 65 | Gorodetskaya et al. ^20^ | BETA |
| Utility value CKD stage 5 | 0·570 | 0·330 | 22 | Lee et al. ^21^ | BETA |
| Utility of dead health state | 0·000 | 0·000 | 0 | Assumption | FIXED |
| Utility value dialysis | 0·440 | 0·320 | 60 | Lee et al. ^21^ | BETA |
| Utility of death due to CVE | 0·000 | 0·000 | 0 | Assumption | FIXED |
| Utility value non-fatal MI | 0·704 | 0·199 | 244 | Sullivan et al. ^22^ | BETA |
| Utility value non-fatal stroke | 0·730 | 0·240 | 352 | Van Eeden et al. ^23^ | BETA |
| Utility value post non-fatal MI | 0·725 | 0·102 | 1211 | Sullivan et al. ^22^ | BETA |
| Utility value post non-fatal stroke | 0·740 | 0·240 | 352 | Sullivan et al. ^22^ | BETA |
| Utility value Transplant: 0-1 year following transplant | 0·710 | 0·270 | 125 | Lee et al. ^21^ | BETA |
| Utility value Transplant: 1-2 year following transplant | 0·710 | 0·270 | 125 | Lee et al. ^21^ | BETA |
| Utility value Transplant: 2-3 year following transplant | 0·710 | 0·270 | 125 | Lee et al. ^21^ | BETA |
| Utility value Transplant: 3+ year following transplant | 0·710 | 0·270 | 125 | Lee et al. ^21^ | BETA |
| Utility general Dutch population - 40-49y | 0·850 | 0·196 | 202 | Versteegh et al. ^19^ | BETA |
| Utility general Dutch population - 50-59y | 0·857 | 0·183 | 186 | Versteegh et al. ^19^ | BETA |
| Utility general Dutch population - 60-69y | 0·839 | 0·179 | 158 | Versteegh et al. ^19^ | BETA |
| Utility general Dutch population - 70y+ | 0·852 | 0·148 | 106 | Versteegh et al. ^19^ | BETA |

CKD=Chronic Kidney Disease. CVE=cardiovascular event. MI=Myocardial Infarction. n_pt=number of observation on which estimate is based. SD=standard deviation. Y=years.

### Costs

#### Costs of screening process

Costs of the home screening (first and confirmation tests), and extensive screening are taken into account. These were based on information from the THOMAS study. Screening costs were separated in costs associated with 1) sending the home tests and 2) costs associated with returning the urine sample and analyzing the urine sample. The confirmatory tests were the same as the first home tests received by participants. Costs of the screening process are provided in euros in the table below. These costs were assumed to be fixed.

**Table S13. Costs associated with the screening process.**

| **Description** | **mean** | **Source** | **Distribution** |
| --- | --- | --- | --- |
| Costs sending screening device (€ 0·9 device, €0·67 safety bag, €2·25 delivery) | 3·82 | Van Mil et al. ^1^ | FIXED |
| Costs returning screening device (€2·25 delivery, €6·13 analysis) | 8·38 | Van Mil et al. ^1^ | FIXED |
| Costs of elaborate screening | 53·8 | Van Mil et al. ^1^ | FIXED |

####

#### Health care costs

The health care costs used in the health economic models are presented in the Table below. Persons who were not diagnosed with kidney function impairment (and who were not already on treatment), were assumed not to incur health care costs related to CKD. All costs are shown as yearly costs, except when described otherwise.

**Table S14. Costs associated with each health state and event.**

| **Description** | **mean** | **SD** | **n_pt** | **Source** | **Distribution** |
| --- | --- | --- | --- | --- | --- |
| Daily costs ACE-inhibitors | 0·04 | 0 | NA | National Health Care Institute ^24^ | FIXED |
| Daily costs combination treatment (ACE-inhibitors, betablockers, diuretic) | 0·16 | 0 | NA | National Health Care Institute ^24^ | FIXED |
| Costs of GP consult after screening | 36 | 0 | NA | National Health Care institute ^25^ | FIXED |
| Yearly HC costs center hemodialysis (used as costs for dialysis) | 100,172 | 23,254 | 3430 | Mohnen et al. ^26^ | GAMMA |
| Yearly costs CKD stage 0 | 0 | 0 | NA | Assumption | FIXED |
| Yearly costs CKD stage 1 | 36 | 0 | NA | 1 control per year at the GP (NHG standards and iMTA costing tool) | FIXED |
| Yearly costs CKD stage 2 | 134 | 0 | NA | 1 control per year at the GP + 1 policlinic appointment (NHG standards and iMTA costing tool) | FIXED |
| Yearly costs CKD stage 3b | 295 | 197-394 | NA | 3 policlinic appointments (NHG standards and iMTA costing tool) | TRIANGULAR |
| Yearly costs CKD stage 3a | 295 | 197-394 | NA | 3 policlinic appointments (NHG standards and iMTA costing tool) | TRIANGULAR |
| Yearly costs CKD stage 4 | 11,929 | 7,034 | 10745 | Oosten et al. ^27^ | GAMMA |
| Yearly costs CKD stage 5 | 11,929 | 7,034 | 10745 | Oosten et al. ^27^ | GAMMA |
| Costs of being dead | 0 | 0 | NA | Assumption | FIXED |
| HC costs of fatal CVD event | 5,621 | 7,731 | 25657 | Soekhlal et al. ^28^ | GAMMA |
| HC costs of non-fatal MI (year 1) | 5,621 | 7,731 | 25657 | Soekhlal et al. ^28^ | GAMMA |
| HC costs of non-fatal stroke (year 1) | 20,226 | 2,369 | 352 | Van Eeden et al. ^23^ | GAMMA |
| HC costs of post non-fatal MI (years >= 2) | 1,268 | 39 | NA | Greving et al. ^29^ | GAMMA |
| HC costs of post non-fatal stroke (years >= 2) | 10,399 | 1,326 | NA | Stam-Slob et al. ^30^ | GAMMA |
| Yearly HC costs transplantation | 92,072 | 42,916 | 2829 | Mohnen et al. ^26^ | GAMMA |
| HC costs year 1 after transplantation | 32,028 | 36,881 | 1825 | Mohnen et al. ^26^ | GAMMA |
| HC costs year 2 after transplantation | 21,801 | 28,739 | 911 | Mohnen et al. ^26^ | GAMMA |
| Yearly HC costs year 3 (and later) after transplantation | 16,243 | 17,507 | 806 | Mohnen et al. ^26^ | GAMMA |
| Daily costs dapagliflozin | 1 | 0 | NA | National Health Care Institute ^24^ | FIXED |
| Daily costs statins | 0·02 | 0 | NA | National Health Care Institute ^24^ | FIXED |

n_pt=number of observation on which estimate is based. SD=standard deviation. ACE-inhibitors=angiotensin-converting enzyme inhibitors. GP=general practitioner. HC=health care. CKD=chronic kidney disease. CVD=cardiovascular disease. MI=myocardial infarction. NA=not applicable.

## Probabilistic analysis details - CVE risk distribution

This section explains how the probabilistic parameters of the CVE risk distributions were obtained. We followed a similar approach as described in Lagerweij et al. ^10^
To obtain probabilistic parameters of the CVE risk distributions, the synthetic cohort was bootstrapped. The bootstrap was stratified by age decades. SCORE2 and its add-on were then applied to these bootstrapped samples to obtain a new CVE risk distribution for each iteration of the probabilistic analysis. New random numbers determining progression and the occurrence of renal and cardiovascular events were drawn for each iteration.

## Model validation

### AdViSHE

General validation effort are reported using AdviSHE. ^31^

### TECH-VER

Technical validation of the health economic model was performed using recommendations from TECH-VER. ^32^ Firstly, technical validation was performed through black-box testing. The following black box tests were performed: *does a shorter time horizon lead to lower total LYs, QALYs, and costs?*, *discount rates set to 0%*, *all utility inputs are set to 1*, *all utility inputs are set to 0*, *all cost inputs are set to 0*, *does the total quality-adjusted life years divided by total life years lies between minimum and maximum utility values?*, and *are discounted results are lower than non-discounted results?*. We also investigated whether probabilistic estimates of costs, rates, relative risk, and hazard ratios were strictly positive and whether probabilistic utility values and probabilities remained between 0 and 1. Additionally, the convergence of the incremental QALYs and costs of home-based screening versus usual care were inspected visually.

# Results

## Validation

### AdViSHE

#### Part A: Validation of the conceptual model

*- A1: Face validity testing (conceptual model): Have experts been asked to judge the appropriateness of the conceptual model?*

Yes, a clinical expert (RG) and a health economist (CB) have been consulted during the development of the health economic model during project team meeting and they have agreed with the final structure and assumptions of the health economic model. RG is considered a clinical expert because he is a Professor of Medicine in the area of Urology & Nephrology. He has dedicated his career to the role of (increased) albuminuria in CKD and is coordinator of the Prevention of Renal and Vascular End-stage Disease (PREVEND). CB is considered a health economic expert in his disease area since he has been involved in multiple research project using the PREVEND and he has assessed the potential cost-effectiveness of screening for albuminuria in a previous health economic analysis. ^7^

*- A2: Cross validity testing (conceptual model): Has this model been compared to other conceptual models found in the literature or clinical textbooks?*

Yes, as in Hoerger et al. CKD staging is defined by the albuminuria level and eGFR level of individuals. ^33^ Albuminuria were defined in a similar fashion as in Boersma et al. and eGFR decline is modelled by a yearly decrease as in Hoerger et al. ^7^ We also included cardiovascular event as in previous health economic models of CKD described in the systematic review of Sugrue et al. since increase albuminuria and CKD are risk factors for the occurrence of cardiovascular events. ^34^ Furthermore, CKD staging in the health current model is based on the recommendations of Dutch general practitioner society.
In addition to other health economic models in this disease area, we also considered the albuminuria-level and eGFR when estimating the risk of cardiovascular events using a recently-developed add-on on the SCORE2 prediction model.

#### Part B: Input data validation

*-B1: Face validity testing (input data): Have experts been asked to judge the appropriateness of the input data?*

Yes, RG has been consulted to determine the plausibility of the relative effectiveness of treatments in the health economic model. The reasons for considering him an expert are described in *Part A*. Based on discussions with the clinical expert (RG), the eGFR decline was stratified by age category (younger than 55 and above 55 years old) instead of using a single eGFR decline per albuminuria category. The sources to inform relative effectiveness estimates of treatments, e.g. ACE inhibition, were discussed and selected based on clinical expert opinion (RG).

*-B2: Model fit testing: When input parameters are based on regression models, have statistical tests been performed?*

Not applicable in this health economic model since we did not develop any regression models (or other statistical models) ourselves.

#### Part C: Validation of the computerized model

*-C1: External review: Has the computerized model been examined by modelling experts?*

No formal review of the health economic model has been performed. However, modelling aspects have been extensively discussed between XP and HK.

*-C2: Extreme value testing: Has the model been run for specific, extreme sets of parameter values in order to detect any coding errors?*

Yes, see the *TECH-VER* section below.

*-C3: Testing of traces: Have patients been tracked through the model to determine whether its logic is correct?*

Yes, but this is not reported in the current report.

*-C4: Unit testing: Have individual sub-modules of the computerized model been tested?*

Yes, but this is not reported in the current report.

#### Part D: Operational validation

*-D1: Face validity testing (model outcomes): Have experts been asked to judge the appropriateness of the model outcomes?*Yes, all authors have been involved in the validation of the results. The involved experts are the ones involved in previous steps.

*-D2: Cross validation testing (model outcomes): Have the model outcomes been compared to the outcomes of other models that address similar problems?*No, this has not been performed due to the limited number of studies focusing on this setting and using an individual-level model (to the best of the authors’ knowledge).

*-D3: Validation against outcomes using alternative input data: Have the model outcomes been compared to the outcomes obtained when using alternative input data? Yes, this has partly been performed in the* scenario and subgroup analyses. The results of these analyses are reported in the manuscript.

*-D4: Validation against empirical data: Have the model outcomes been compared to empirical data?*No, this has not been performed.

### TECH-VER

During development, black box testing revealed that the QALY calculation did not take account that individuals were alive. Hence, QALY accrued for dead individuals, this has been corrected (module calculating effects and costs) during development. In a module of the model, there were references to objects which were not defined at the start of the module, which led to errors. All black box tests results in the expected results and all probabilistic input parameters lied within the expected range: costs, rates, relative risks and hazard ratios were strictly positive, and probabilities and utility values had values between 0 and 1.

Originally, the results of the scenario analyses including treatment discontinuation were not highly different from the results of the base-case analysis. Upon model review, it was discovered that adherence was not correctly implemented, which has been adapted for the final analysis.

**Table S15. TECH-VER checks.**

| **Check** | **Expectation** | **Expectation met** |
| --- | --- | --- |
| Do the total life-years, QALYs, and costs decrease if a shorter time horizon is selected? | Shorter time horizon leads to lower total undiscounted life years, quality-adjusted life years, and costs | Yes |
| No discount on effects and costs | Discounted and undiscounted effects and costs are equal | Yes |
| All utility inputs are set to 1 | All total quality-adjusted life years are equal to total life years | Yes |
| All utility inputs are set to 0 | All total quality-adjusted life years are 0 | Yes |
| All cost inputs are set to 0 | All total costs are 0 | Yes |
| Total quality-adjusted life years divided by total life years | Results should lie between min and max utility value | Yes |
| Discounted results are lower than non-discounted results |  | Yes |

QALY=quality of life.

#### Convergence of the incremental QALYs and costs

Here below, the convergence plots of the incremental QALYs and costs of home-based screening versus usual care can be seen.

**Figure S7. Convergence plots average quality of life (A) and average costs (B).**


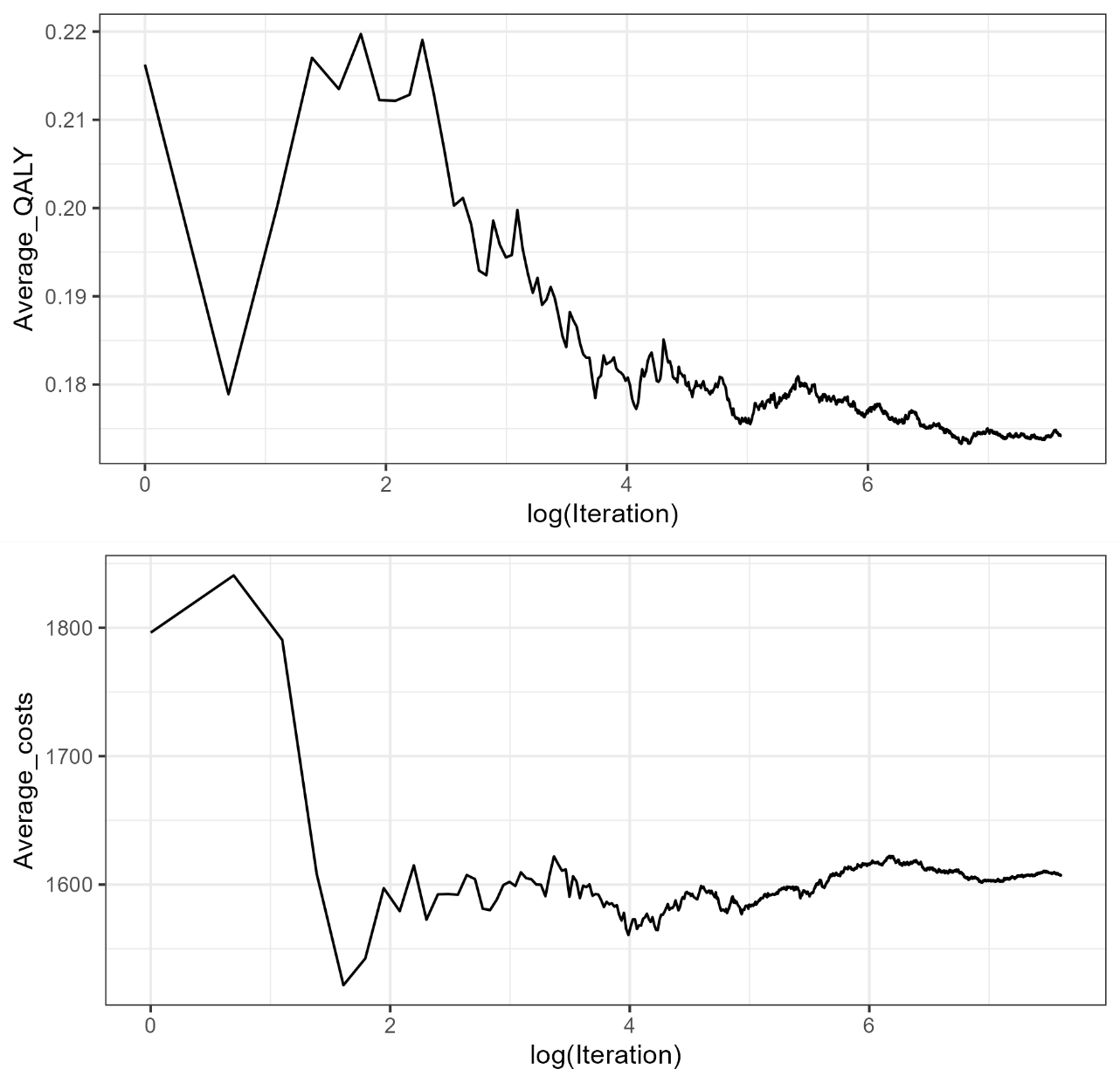
QALY=quality of life.

A

B

## Events over time

The following plots show the proportion of individuals experiencing outcomes (cardiovascular events, dialysis, transplantation, or death) during each cycle of the health economic model. The proportion is calculated as the number of individuals experiencing the event per cycle divided by the total number of individuals who entered the simulation.

**Figure S8. Proportion of individuals experiencing an event.**

CKD = Chronic Kidney Disease. CVE = Cardiovascular Event. MI = Myocardial Infarction. UC = Usual Care. UCD = Urine Collection Device (home-based screening).


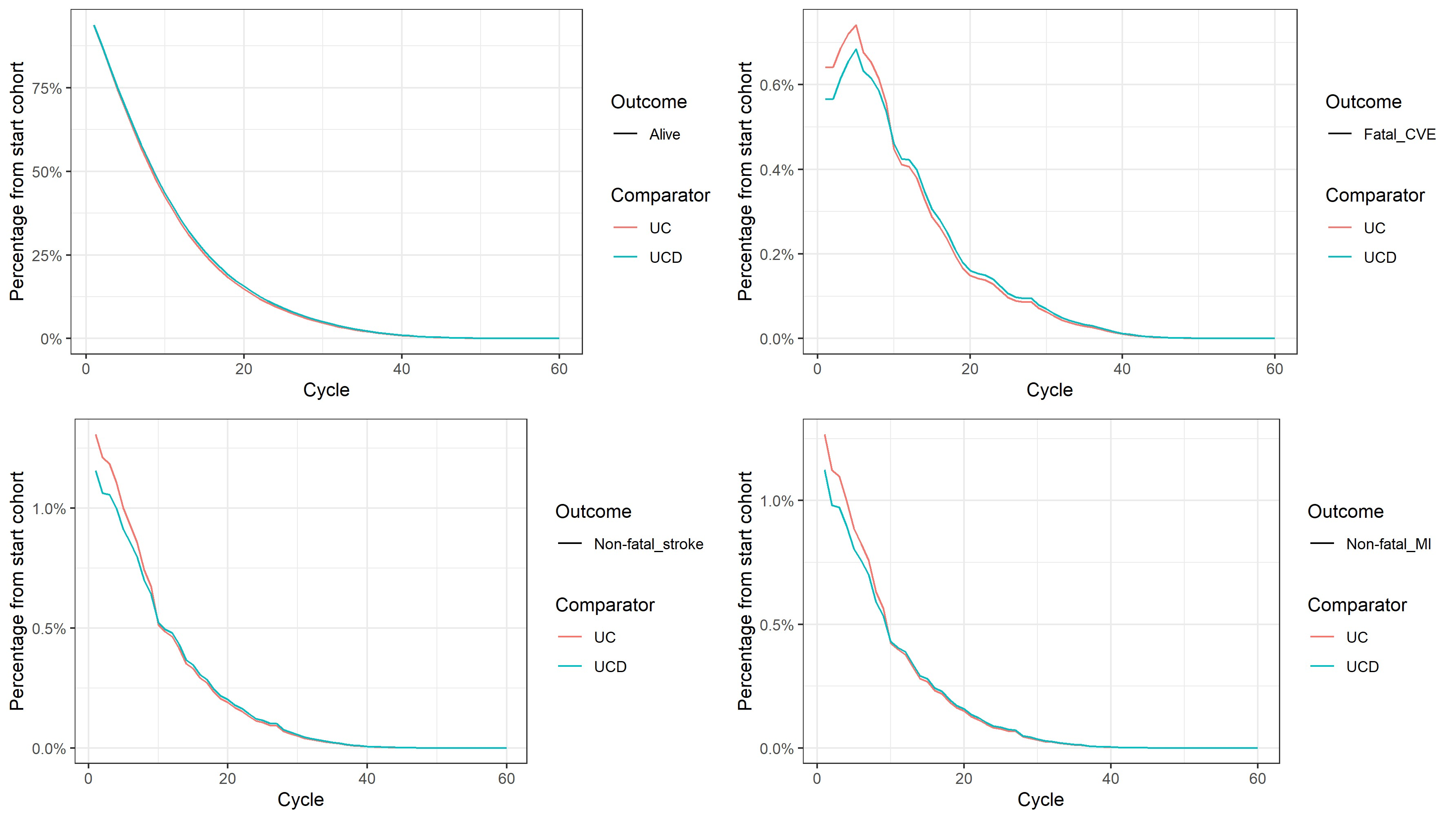


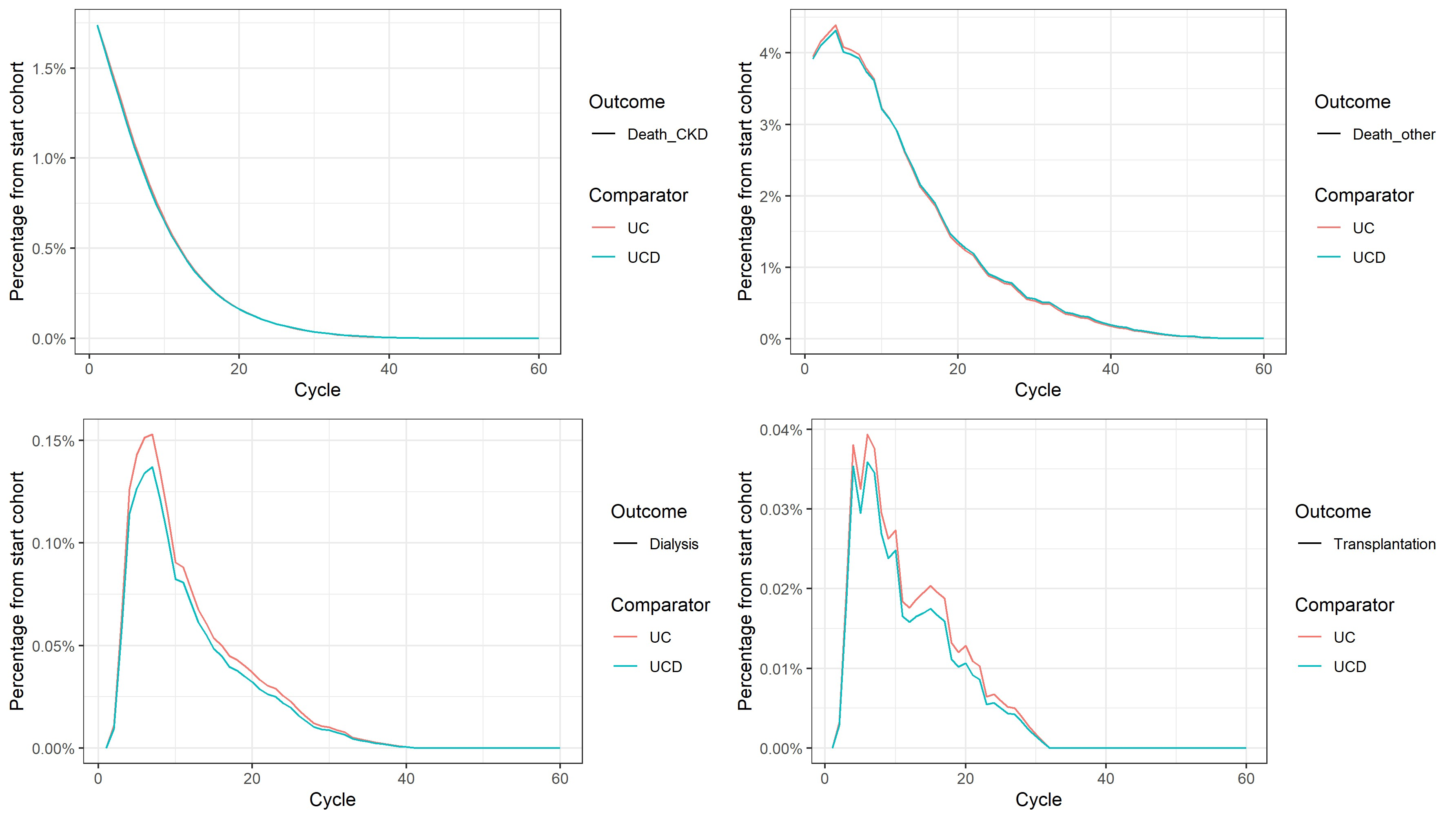


## Event rates and lifetime probability of kidney and cardiovascular events for scenario analysis 5

The following table shows the number and probability of kidney and cardiovascular events per strategy, expressed per 100.000 individuals participating in the elaborate screening after identification of albuminuria by home-based screening – depicted for scenario analysis 5 (all individuals with known risk factors, outside target values, and newly-diagnosed risk factors went to the GP after referral and experienced full treatment effectiveness).

**Table S16. Number and probability of kidney and cardiovascular events per strategy, expressed per 100.000 individuals participating in the elaborate screening after identification of albuminuria by home-based screening – depicted for scenario 5: treatment optimization after referral.**

|  | **Usual care** | **Screening** | **Absolute difference** | **Relative difference** |
| --- | --- | --- | --- | --- |
| **Non-fatal myocardial infarction** | | | | |
| N | 12,080 | 10,903 | -1,177 | -9·7% |
| Probability | 12·1% | 10·9% | -1·2% |  |
| **Non-fatal stroke** | | | | |
| N | 14,035 | 12,909 | -1126 | -8.0·% |
| Probability | 14·0% | 12·9% | -1·1% |  |
| **Fatal cardiovascular event** | | | | |
| N | 10,541 | 10,163 | -378 | -3·6% |
| Probability | 10·5% | 10·2% | -0·40% |  |
| **Dialysis** | | | | |
| N | 1,799 | 1,467 | -332 | -18·5% |
| Probability | 1·8% | 1·5% | -0·33% |  |
| **Kidney transplantation** | | | | |
| N | 485 | 393 | -92 | -19·0% |
| Probability | 0·48% | 0·39% | -0·092% |  |

N= number.

## One-year budget impact analysis home-based screening versus usual care – screening all 45-80 year old individuals in the Netherlands in 2021 (n = 7,545,845)

The following table show the detailed one-year budget impact analysis.

**Table S17. One-year budget impact of the home-based albuminuria screening using home-based screening versus usual care, in case all 45-80 year old individuals in the Netherlands in 2021 were screened (n = 7,545,845), based on costs, participation rates and yield of screening in THOMAS.**

|  | **Individuals usual care (n) *** | **Individuals screening (n)*** | **Costs per individual** | **Total costs usual care*** | **Total costs screening*** | **BI screening vs· usual care*** |
| --- | --- | --- | --- | --- | --- | --- |
| **Cost item of home-based albuminuria screening** |  |  |  |  |  |  |
| **Home-based screening** |  |  |  |  |  |  |
| Screening device + delivery to individual | - | 7,545,845 | € 3·82 | - | € 28,825,128 | € 28,825,128 |
| Screening device | - | 7,545,845 | € 1·57 | - | € 11,846,977 | € 11,846,977 |
| Delivery to individual | - | 7,545,845 | € 2·25 | - | € 16,978,151 | € 16,978,151 |
| Return screening device + analysis in laboratory | - | 4,503,327 | € 8·38 | - | € 37,737,878 | € 37,737,878 |
| Return to laboratory | - | 4,503,327 | € 2·25 | - | € 10,132,485 | € 10,132,485 |
| Analysis in laboratory | - | 4,503,327 | € 6·13 | - | € 27,605,393 | € 27,605,393 |
| Confirmatory screening device + delivery to individual | - | 238,805 | € 3·82 | - | € 912,236 | € 912,236 |
| Screening device | - | 238,805 | € 1·57 | - | € 374,924 | € 374,924 |
| Delivery to individual | - | 238,805 | € 2·25 | - | € 537,312 | € 537,312 |
| Return confirmatory screening device + analysis in laboratory | - | 221,819 | € 8·38 | - | € 1,858,844 | € 1,858,844 |
| Return to laboratory | - | 221,819 | € 2·25 | - | € 499,093 | € 499,093 |
| Analysis in laboratory | - | 221,819 | € 6·13 | - | € 1,359,751 | € 1,359,751 |
| Confirmatory screening device 2 + delivery to individual | - | 99,918 | € 3·82 | - | € 381,689 | € 381,689 |
| Screening device | - | 99,918 | € 1·57 | - | € 156,872 | € 156,872 |
| Delivery to individual | - | 99,918 | € 2·25 | - | € 224,817 | € 224,817 |
| Return confirmatory screening device 2 + analysis in laboratory | - | 92,695 | € 8·38 | - | € 776,788 | € 776,788 |
| Return to laboratory | - | 92,695 | € 2·25 | - | € 208,565 | € 208,565 |
| Analysis in laboratory | - | 92,695 | € 6·13 | - | € 568,223 | € 568,223 |
| **Elaborate screening** |  |  |  |  |  |  |
| Elaborate screening of positive individuals | - | 125,760 | € 53·8 | - | € 6,765,883 | € 6,765,883 |
| **Treatment optimization** |  |  |  |  |  |  |
| Visiting general practitioner | - | 66,843 | € 35·7 | - | € 2,385,775 | € 2,385,775 |
| Starting or continuing treatment | - | 98,820 | € 442** | € 24,268,764 | € 43,836,625 | € 19,567,861 |
| **Budget impact** |  |  |  |  |  |  |
| Total budget impact | - | - | - | - | - | € 99,212,082 |
| Total BI screening | - | - | - | - | - | € 70·492·563,00 (71·1%) |
| Total BI screening device | - | - | - | - | - | € 12·378·773,00 (12·5%) |
| Total BI delivery | - | - | - | - | - | € 28·580·423,00 (28·8%) |
| Total BI analysis | - | - | - | - | - | € 29·533·367,00 (29·9%) |
| Total BI elaborate screening | - | - | - | - | - | € 6,765,883 (6·8%) |
| Total BI treatment (implementation of care) | - | - | - | - | - | € 21·953·636 (22·1%) |
| BI = budget impact. GP = General Practitioner. N = number. * Rounded **Rounded weighted average of yearly treatment costs using both strategies. | | | | | | |

# R session information

sessionInfo()

## R version 4.2.2 (2022-10-31 ucrt)
## Platform: x86_64-w64-mingw32/x64 (64-bit)
## Running under: Windows 10 x64 (build 22621)
##
## Matrix products: default
##
## locale:
## [1] LC_COLLATE=Dutch_Netherlands.utf8 LC_CTYPE=Dutch_Netherlands.utf8
## [3] LC_MONETARY=Dutch_Netherlands.utf8 LC_NUMERIC=C
## [5] LC_TIME=Dutch_Netherlands.utf8
##
## attached base packages:
## [1] stats graphics grDevices utils datasets methods base
##
## other attached packages:
## [1] forcats_0.5.1 stringr_1.5.0 dplyr_1.1.0
## [4] purrr_1.0.1 readr_2.1.3 tidyr_1.3.0
## [7] tibble_3.0.5 ggplot2_3.4.1 tidyverse_1.3.0
## [10] knitr_1.42 THOMAShemod_0.0.0.9000 testthat_3.1.7
##
## loaded via a namespace (and not attached):
## [1] fs_1.5.2 usethis_2.1.6 lubridate_1.7.9.2
## [4] devtools_2.4.5 httr_1.4.5 rprojroot_2.0.2
## [7] tools_4.2.2 profvis_0.3.7 backports_1.2.0
## [10] utf8_1.1.4 R6_2.5.0 rriskDistributions_2.1.2
## [13] DBI_1.1.1 colorspace_2.0-0 urlchecker_1.0.1
## [16] withr_2.5.0 tidyselect_1.2.0 prettyunits_1.1.1
## [19] processx_3.8.0 compiler_4.2.2 cli_3.6.0
## [22] rvest_0.3.6 expm_0.999-6 xml2_1.3.3
## [25] desc_1.4.2 scales_1.2.1 mvtnorm_1.1-1
## [28] mc2d_0.1-18 callr_3.7.3 digest_0.6.31
## [31] rmarkdown_2.20 pkgconfig_2.0.3 htmltools_0.5.4
## [34] sessioninfo_1.2.2 dbplyr_2.0.0 fastmap_1.1.0
## [37] htmlwidgets_1.5.3 rlang_1.1.0 readxl_1.3.1
## [40] rstudioapi_0.13 shiny_1.7.4 generics_0.1.0
## [43] jsonlite_1.7.2 magrittr_2.0.1 Matrix_1.2-18
## [46] Rcpp_1.0.8.3 munsell_0.5.0 fansi_0.4.2
## [49] lifecycle_1.0.3 stringi_1.5.3 yaml_2.2.1
## [52] brio_1.1.1 MASS_7.3-53 pkgbuild_1.4.0
## [55] grid_4.2.2 promises_1.2.0.1 crayon_1.3.4
## [58] miniUI_0.1.1.1 lattice_0.20-41 haven_2.3.1
## [61] splines_4.2.2 hms_1.0.0 ps_1.5.0
## [64] pillar_1.8.1 eha_2.8.5 pkgload_1.3.2
## [67] reprex_1.0.0 glue_1.6.2 evaluate_0.20
## [70] msm_1.6.8 remotes_2.4.2 modelr_0.1.8
## [73] vctrs_0.6.0 tzdb_0.3.0 httpuv_1.6.7
## [76] cellranger_1.1.0 gtable_0.3.0 assertthat_0.2.1
## [79] cachem_1.0.6 xfun_0.37 mime_0.9
## [82] xtable_1.8-4 broom_1.0.4 pracma_2.3.3
## [85] later_1.3.0 survival_3.2-7 memoise_2.0.1
## [88] writexl_1.4.0 fitdistrplus_1.1-3 ellipsis_0.3.2

# References

1 van Mil D, Kieneker LM, Evers-Roeten B, *et al.* Participation rate and yield of two home-based screening methods to detect increased albuminuria in the general population in the Netherlands (THOMAS): a prospective, randomised, open-label implementation study. *The Lancet* 2023. DOI:https://doi.org/10.1016/S0140-6736(23)00876-0.

2 Nowok B, Raab GM, Dibben C. synthpop: Bespoke Creation of Synthetic Data in R. *J Stat Softw* 2016; **74**: 1–26.

3 Kidney disease: Improving global outcomes (KDIGO) CKD work group. KDIGO 2012 clinical practice guideline for the evaluation and management of chronic kidney disease. *Kidney Int Suppl (2011)* 2013; **3**: 1–150.

4 Brantsma AH, Atthobari J, Bakker SJL, *et al.* What Predicts Progression and Regression of Urinary Albumin Excretion in the Nondiabetic Population? *Journal of the American Society of Nephrology* 2007; **18**: 637–45.

5 Matsushita K, Kaptoge S, Hageman SHJ, *et al.* Including measures of chronic kidney disease to improve cardiovascular risk prediction by SCORE2 and SCORE2-OP. *Eur J Prev Cardiol* 2023; **30**: 5–16.

6 Centraal Bureau voor de Statistiek. Statline. https://opendata.cbs.nl (accessed Dec 7, 2022).

7 Boersma C, Gansevoort RT, Pechlivanoglou P, *et al.* Screen-and-treat strategies for albuminuria to prevent cardiovascular and renal disease: cost-effectiveness of nationwide and targeted interventions based on analysis of cohort data from the Netherlands. *Clin Ther* 2010; **32**: 1103–21.

8 Fleurence RL, Hollenbeak CS. Rates and Probabilities in Economic Modelling. *Pharmacoeconomics* 2007; **25**: 3–6.

9 van der Velde M, Halbesma N, de Charro FT, *et al.* Screening for albuminuria identifies individuals at increased renal risk. *J Am Soc Nephrol* 2009; **20**: 852–62.

10 Lagerweij GR, Brouwers L, De Wit GA, *et al.* Impact of preventive screening and lifestyle interventions in women with a history of preeclampsia: A micro-simulation study. *Eur J Prev Cardiol* 2020; **27**: 1389–99.

11 Baigent C, Landray MJ, Reith C, *et al.* The effects of lowering LDL cholesterol with simvastatin plus ezetimibe in patients with chronic kidney disease (Study of Heart and Renal Protection): a randomised placebo-controlled trial. *The Lancet* 2011; **377**: 2181–92.

12 Wei J, Galaviz KI, Kowalski AJ, *et al.* Comparison of Cardiovascular Events among Users of Different Classes of Antihypertension Medications: A Systematic Review and Network Meta-analysis. *JAMA Netw Open* 2020; **3**. DOI:10.1001/jamanetworkopen.2019.21618.

13 Taylor F, Huffman MD, Macedo AF, *et al.* Statins for the primary prevention of cardiovascular disease. *Cochrane Database of Systematic Reviews* 2013. DOI:10.1002/14651858.CD004816.pub5.

14 Heerspink HJL, Stefánsson B V, Correa-Rotter R, *et al.* Dapagliflozin in Patients with Chronic Kidney Disease. *New England Journal of Medicine* 2020; **383**: 1436–46.

15 Chronic Kidney Disease Prognosis Consortium, Matsushita K, van der Velde M, *et al.* Association of estimated glomerular filtration rate and albuminuria with all-cause and cardiovascular mortality in general population cohorts: a collaborative meta-analysis. *Lancet* 2010; **375**: 2073–81.

16 Neovius M, Jacobson SH, Eriksson JK, Elinder C-G, Hylander B. Mortality in chronic kidney disease and renal replacement therapy: a population-based cohort study. *BMJ Open* 2014; **4**: e004251.

17 Manns B, Hemmelgarn B, Tonelli M, *et al.* Population based screening for chronic kidney disease: cost effectiveness study. *BMJ* 2010; **341**: c5869.

18 Okano Y, Tamura K, Masuda S, Ozawa M, Tochikubo O, Umemura S. Effects of Angiotensin II Receptor Blockers on the Relationships Between Ambulatory Blood Pressure and Anti-Hypertensive Effects, Autonomic Function, and Health-Related Quality of Life. *Clin Exp Hypertens* 2009; **31**: 680–9.

19 M. Versteegh M, M. Vermeulen K, M. A. A. Evers S, de Wit GA, Prenger R, A. Stolk E. Dutch Tariff for the Five-Level Version of EQ-5D. *Value in Health* 2016; **19**: 343–52.

20 Gorodetskaya I, Zenios S, Mcculloch CE, *et al.* Health-related quality of life and estimates of utility in chronic kidney disease. *Kidney Int* 2005; **68**: 2801–8.

21 Lee AJ, Morgan CL, Conway P, Currie CJ. Characterisation and comparison of health-related quality of life for patients with renal failure. *Curr Med Res Opin* 2005; **21**. DOI:10.1185/030079905X65277.

22 Sullivan PW, Lawrence WF, Ghushchyan V. A national catalog of preference-based scores for chronic conditions in the United States. *Med Care* 2005; **43**. DOI:10.1097/01.mlr.0000172050.67085.4f.

23 Van Eeden M, Van Heugten C, Van Mastrigt GAPG, Van Mierlo M, Visser-Meily JMA, Evers SMAA. The burden of stroke in the Netherlands: Estimating quality of life and costs for 1 year poststroke. *BMJ Open* 2015; **5**. DOI:10.1136/bmjopen-2015-008220.

24 National Health Care Institute. Medicijnkosten.nl. https://www.medicijnkosten.nl/ (accessed Dec 7, 2022).

25 National Health Care Institute. Richtlijn voor het uitvoeren van economische evaluaties in de gezondheidszorg. 2016 https://www.zorginstituutnederland.nl/over-ons/publicaties/publicatie/2016/02/29/richtlijn-voor-het-uitvoeren-van-economische-evaluaties-in-de-gezondheidszorg (accessed Dec 7, 2022).

26 Mohnen SM, van Oosten MJM, Los J, *et al.* Healthcare costs of patients on different renal replacement modalities – Analysis of Dutch health insurance claims data. *PLoS One* 2019; **14**. DOI:10.1371/journal.pone.0220800.

27 van Oosten MJM, Logtenberg SJJ, Leegte MJH, *et al.* Age-related difference in health care use and costs of patients with chronic kidney disease and matched controls: Analysis of Dutch health care claims data. *Nephrology Dialysis Transplantation* 2021; **35**: 2138–46.

28 Soekhlal RR, Burgers LT, Redekop WK, Tan SS. Treatment costs of acute myocardial infarction in the Netherlands. *Netherlands Heart Journal* 2013; **21**: 230–5.

29 Greving JP, Visseren FLJ, de Wit GA, Algra A. Statin treatment for primary prevention of vascular disease: Whom to treat? Cost-effectiveness analysis. *BMJ* 2011; **342**. DOI:10.1136/bmj.d1672.

30 Stam-Slob MC, van der Graaf Y, de Boer A, Greving JP, Visseren FLJ. Cost-effectiveness of PCSK9 inhibition in addition to standard lipid-lowering therapy in patients at high risk for vascular disease. *Int J Cardiol* 2018; **253**: 148–54.

31 Vemer P, Corro Ramos I, van Voorn GAK, Al MJ, Feenstra TL. AdViSHE: A Validation-Assessment Tool of Health-Economic Models for Decision Makers and Model Users. *Pharmacoeconomics* 2016; **34**: 349–61.

32 Büyükkaramikli NC, Rutten-van Mölken MPMH, Severens JL, Al M. TECH-VER: A Verification Checklist to Reduce Errors in Models and Improve Their Credibility. *Pharmacoeconomics* 2019; **37**: 1391–408.

33 Hoerger TJ, Wittenborn JS, Segel JE, *et al.* A Health Policy Model of CKD: 1. Model Construction, Assumptions, and Validation of Health Consequences. *American Journal of Kidney Diseases* 2010; **55**: 452–62.

34 Sugrue DM, Ward T, Rai S, McEwan P, van Haalen HGM. Economic Modelling of Chronic Kidney Disease: A Systematic Literature Review to Inform Conceptual Model Design. Pharmacoeconomics. 2019; **37**: 1451–68.
